# Cardiac Complications of Infections Due to Respiratory Syncytial Virus and Human Metapneumovirus: A Systematic Review and Pooled Analysis of Clinical Features

**DOI:** 10.3390/v18101106

**Published:** 2026-10-07

**Authors:** Matteo Riccò, Luca Pipitò, Claudio Costantino, Marco Bottazzoli, Paolo Manzoni, Marco Falcone, Pasquale Gianluca Giuri, Antonio Cascio

**Affiliations:** 1 Servizio di Prevenzione e Sicurezza Negli Ambienti di Lavoro (SPSAL), AUSL–IRCCS di Reggio Emilia, 42122 Reggio Emilia, Italy; 2 Infectious and Tropical Diseases Unit, Department of Health Promotion, Mother and Child Care, Internal Medicine and Medical Specialties, “G. D’Alessandro”, University of Palermo, AOUP P. Giaccone, 90127 Palermo, Italy; luca.pipito@community.unipa.it (L.P.); antonio.cascio03@unipa.it (A.C.); 3 Department of Health Promotion Sciences, Maternal and Infant Care, Internal Medicine and Medical Specialties (PROMISE) “G. D’Alessandro”, University of Palermo, AOUP P. Giaccone, 90127 Palermo, Italy; claudio.costantino01@unipa.it; 4 Department of Otorhinolaryngology, ASUIT (Azienda Sanitaria Universitaria Integrata del Trentino) Trento, 38122 Trento, Italy; marco.bottazzoli@asuit.tn.it; 5 SCDU Pediatria e Neonatologia, Ospedale Degli Infermi, Ponderano (BI), Dipartimento Materno-Infantile ASLBI, Università di Torino, 10124 Torino, Italy; p.manzoni@unito.it; 6 Department of Clinical and Experimental Medicine, University of Pisa, 56126 Pisa, Italy; marco.falcone@unipi.it; 7 Department of Medicine and Diagnostics, AUSL (Azienda Unità Sanitaria Locale) di Parma, 43100 Parma, Italy; pgiuri@ausl.pr.it

**Keywords:** respiratory syncytial virus, human metapneumovirus, myocarditis, cardiac complications, arrhythmias

## Abstract

Respiratory syncytial virus (RSV) and human metapneumovirus (hMPV) are major causes of respiratory tract infections across different age groups. Although primarily regarded as respiratory pathogens, both viruses have been associated with cardiac complications, whose clinical spectrum remains incompletely characterized. We systematically reviewed published cases of RSV- and hMPV-associated cardiac involvement and compared their clinical characteristics and outcomes. PubMed, Scopus, and EMBASE were systematically searched for reports describing cardiac complications associated with laboratory-confirmed RSV or hMPV infection. Individual patient data were pooled and analyzed using descriptive and univariate analyses. Multivariable logistic regression was used to identify characteristics associated with hMPV versus RSV diagnosis, while multiple correspondence analysis (MCA) was performed to explore multivariate patterns of clinical presentation. Age-stratified sensitivity analyses were additionally performed. Overall, 102 cases were included, comprising 71 RSV (69.6%) and 31 hMPV (30.4%). hMPV cases were significantly older than RSV cases (41.5 years, interquartile range 3.5–63.0 vs. 1.5 years, interquartile range 0.2–31.0; *p* < 0.001) and had a higher burden of comorbidities. Myocarditis was the most frequently reported cardiac complication in both groups (87.1% hMPV vs. 74.6% RSV), followed by heart failure and pericarditis. ICU admission was more frequent among hMPV cases (96.8% vs. 77.5%; *p* = 0.034), whereas overall clinical outcomes did not significantly differ between groups. In exploratory multivariable analyses, dyspnea (aOR 16.280, 95% CI 1.370–193.475) and cough (aOR 6.059, 95% CI 1.062–34.552) were associated with hMPV diagnosis, whereas rhinorrhea (aOR 0.031, 95% CI 0.003–0.286) and lethargy/altered mental status (aOR 0.103, 95% CI 0.015–0.712) were associated with RSV diagnosis. ICU admission was also associated with hMPV diagnosis (aOR 11.033, 95% CI 1.125–108.173). These estimates were characterized by wide confidence intervals and should be interpreted cautiously. MCA showed substantial overlap between RSV and hMPV cases, without clear separation of their overall clinical presentation, while most between-group differences were attenuated in age-stratified analyses. In conclusion, both RSV and hMPV can be associated with a broad spectrum of potentially severe cardiac complications, with myocarditis representing the predominant reported manifestation. Despite some differences in demographic and clinical characteristics, their overall cardiac and clinical phenotypes substantially overlap. These findings support greater awareness of potential cardiac involvement during severe RSV and hMPV infections, while highlighting the need for prospective, systematically investigated cohorts to establish the incidence, mechanisms, and long-term outcomes of these complications.

## 1. Introduction

Respiratory syncytial virus (RSV) and human metapneumovirus (hMPV) are two closely related, small, enveloped, negative-sense, single-stranded RNA viruses belonging to the *Pneumoviridae* family [1,2,3,4]. The two pathogens share several similarities in genome organization, despite differences in genome size (approximately 13 kb for hMPV and 15.2 kb for RSV) [2,4,5], as well as in viral structure and life cycle, host interactions, and immune-evasion strategies [1,4,5,6]. RSV and hMPV are among the major causes of lower respiratory tract infections (LRTIs) [7,8,9,10,11], including bronchiolitis and viral pneumonia, across all age groups [2,12,13,14,15,16]. The global burden of RSV is substantial, accounting for approximately 3.6 million hospitalizations among infants and children each year [10,11,17]. Estimates for adults aged 18–65 years and older adults aged >65 years are affected by greater uncertainty. Nevertheless, RSV has been estimated to account for 5–15% of acute respiratory infections requiring medical care [18] and represents a major cause of morbidity and mortality among older adults [4,19,20,21]. hMPV similarly accounts for approximately 5–10% of pediatric hospitalizations due to LRTIs [22]. Its epidemiology is more heterogeneous across older age groups, however, with a second peak in incidence among older individuals, resulting in a characteristic U-shaped age distribution [1,5,7,9,17,23,24]. RSV and hMPV also share similar transmission patterns, spreading primarily through respiratory droplets and contact with contaminated surfaces. Both viruses cause seasonal epidemics characterized by high transmissibility and recurrent infections throughout life [4,7,17,20,25,26].

Similar to other respiratory pathogens such as influenza virus [27], emerging evidence suggests that RSV and hMPV may impose a substantial cardiovascular burden [27,28], including myocardial infarction and stroke [29,30,31], particularly among older adults [32,33]. For example, a retrospective cohort study from Canada observed that up to 22% of adults hospitalized for RSV infection experienced cardiovascular complications, including heart failure (14%), arrhythmia (8%), stroke (2%), and myocardial infarction [34]. In their recent report on 2558 adults aged ≥ 65 years hospitalized with RSV infection, Verschoor et al. [33] similarly documented high rates of adverse clinical outcomes, including prolonged hospital stay, ICU admission, and 30-day mortality, compared with patients hospitalized for influenza, urinary tract infection, or fractures. Moreover, the risk of heart failure up to 1 year after discharge was significantly higher regardless of pre-existing cardiovascular conditions (Hazard Ratio [HR] 1.65, 95% Confidence Interval [95% CI] 1.14 to 2.38 compared with influenza), as was the risk of rhythm disorders, particularly atrial fibrillation (HR 1.50, 95% CI 1.08 to 2.08 compared with patients hospitalized for fractures).

Indeed, RSV and hMPV have been associated with a broad spectrum of cardiac manifestations, with direct viral involvement documented in some cases, ranging from myocarditis [35,36,37,38,39,40], pericardial effusion with cardiac tamponade [41], and rhythm disturbances such as supraventricular tachycardia, multifocal atrial tachycardia and atrial flutter, ventricular tachycardia, ventricular fibrillation and, more rarely, slow junctional escape rhythm and advanced-degree atrioventricular block [42]. However, the underlying mechanisms remain poorly understood and are largely hypothetical [27].

Given the potential significance of RSV- and hMPV-associated cardiac complications, a systematic review synthesizing available reports on associated clinical features may be useful to healthcare professionals involved in the management and follow-up of patients with suspected or documented RSV or hMPV infection and cardiac involvement. Moreover, systematic characterization of these cardiac complications may improve our understanding of their clinical spectrum and potentially provide insights into their underlying pathophysiology. Therefore, we designed the present systematic review and pooled analysis of available case reports describing RSV- and hMPV-associated cardiac disease in children and adults, focusing on clinical manifestations, outcomes, and factors associated with complications, and aiming to characterize the distribution of findings across published cases.

## 2. Materials and Methods

### 2.1. Objective and Research Concept

#### 2.1.1. Objective

The objective of this study was to summarize the currently available evidence on short- and long-term cardiac outcomes associated with documented cardiac complications of hMPV and/or RSV infection. We therefore undertook a systematic review in accordance with the PRISMA statement (Preferred Reporting Items for Systematic Reviews and Meta-Analysis) [43,44]. The study was conducted in accordance with a prospectively registered protocol, which is available in the Prospective Register of Systematic Reviews (PROSPERO) database under registration number CRD420251170706 [43,44] (Supplementary File S1; https://www.crd.york.ac.uk/prospero/; accessed on 20 August 2026) [45].

#### 2.1.2. Research Concept

The research concept was preliminarily defined by means of the “PICO” strategy (Patient/Population/Problem; Investigated results; Control/Comparator; Outcome) [46,47], as follows:

- population: patients with laboratory-confirmed RSV or hMPV infection and a reported cardiac manifestation;
- exposure: viral infection, analyzed separately as RSV and hMPV;
- comparator: descriptive comparison of reported RSV and hMPV cases, not an unexposed control group;
- outcomes: predefined cardiac manifestations, acute supportive care, in-hospital outcomes, and follow-up status.

### 2.2. Research Strategy

From 20 August 2024 onward, three scientific databases (i.e., PubMed, Scopus, and Embase) were searched for reports of cardiac complications associated with RSV or hMPV infection in adults and children, with no restriction on publication date (last inquiry: 14 August 2026). The search was supplemented by a snowballing approach, consisting of manual screening of the reference lists of retrieved articles to identify additional eligible reports. The detailed search strategy is reported in Table 1.

### 2.3. Selection Criteria

All primary studies with an observational design (i.e., case reports, case series, cross-sectional studies, and case–control studies) were considered suitable for inclusion in the present systematic review if published in any of the languages understood by the authors (i.e., English, Italian, German, French, Russian, Spanish, Portuguese).

In accordance with the PRISMA guidelines, all potentially eligible records identified through the initial search were screened based on their titles and abstracts [43,44]. If considered consistent with the aims and eligibility criteria of the present review, the full texts of potentially eligible reports were independently assessed by two reviewers.

### 2.4. Investigated Results and Outcomes

The present systematic review included only observational studies, irrespective of design, provided that the reporting strategy allowed for the individual characterization of documented cases. When the same case was reported in multiple publications, the corresponding reports were carefully cross-checked to obtain the most comprehensive description of the clinical case while avoiding duplicate inclusion in the pooled sample. All studies were required to report cardiac disorders in patients with laboratory-confirmed diagnosis of RSV and/or hMPV infection. In both cases, infection was defined as laboratory confirmation of RSV and/or hMPV in respiratory and/or cardiac specimens using one or more of the following diagnostic options:

(a) direct/indirect immunofluorescence (dIF/iIF) on cardiac and/or respiratory specimens;
(b) ELISA on cardiac and/or respiratory specimens;
(c) immunohistochemical testing on cardiac and/or respiratory specimens;
(d) viral isolation on cell cultures;
(e) real-time quantitative polymerase chain reaction (RT-qPCR) on blood and/or cardiac and/or respiratory specimens;
(f) real-time loop-mediated isothermal amplification (RT-LAMP) on blood and/or cardiac and/or respiratory specimens.

Working definitions for the investigated variables and outcomes are summarized in Appendix A, Table A2.

Retrieved studies were excluded from the analyses:

(a) If the full text was only available in a language other than English, Italian, German, French, Russian, Spanish or Portuguese;
(b) If information regarding clinical features and outcomes was insufficient;
(c) If the reported cases of RSV or hMPV infection lacked laboratory confirmation;
(d) If clinical criteria supporting the diagnosis of the underlying cardiac disease were not provided.

### 2.5. Data Extraction

Data extracted by the reviewers included, where available:

(a) Study characteristics: year, month or season, geographic region;
(b) Age and gender of the reported cases;
(c) History of prematurity;
(d) Clinical characteristics at symptom onset. More specifically, the following signs and symptoms were considered: fever (body temperature >38.0 °C); cough; wheezing; dyspnea and/or tachypnea; diagnosis of or symptoms associated with URTI (i.e., signs and symptoms involving the nose, sinuses, and throat, including stuffy or runny nose, sore throat, and cough without documented involvement of bronchi, bronchioles, or alveoli) and/or LRTI status (i.e., bronchitis, bronchiolitis, and pneumonia) and/or ILI (i.e., acute respiratory infection with fever and cough with onset within the previous 10 days) [4,48,49] (see Appendix A, Table A1 for the working definition of investigated conditions). Heart rate and blood pressure values were independently classified according to age-specific reference values derived from currently available reference ranges [50,51]. Tachycardia and bradycardia based on heart rate measurements were considered separately from documented rhythm abnormalities. Sinus tachycardia was therefore not interpreted per se as evidence of a pathological tachyarrhythmia, whereas specific arrhythmias (e.g., supraventricular tachycardia, atrial fibrillation/flutter, ventricular tachycardia, and conduction abnormalities) were classified only when explicitly documented in the original report and/or supported by the reported ECG findings.
(e) Cardiac diagnoses were classified according to the diagnostic terminology and supporting clinical, instrumental, imaging, and/or histopathological findings reported in the original publications. A diagnosis of myocarditis based on clinical or imaging findings was distinguished from direct evidence of viral myocardial involvement, which required identification of RSV or hMPV in cardiac tissue. Detection of the virus in respiratory specimens alone was not considered evidence of direct myocardial infection.
(f) The Charlson Comorbidity Index (CCI) was retrospectively derived from the clinical information available in the original reports to provide a standardized summary measure of the burden of reported underlying conditions across a highly heterogeneous population [52]. The CCI was used primarily as a pragmatic tool to facilitate comparison across cases rather than as a validated prognostic instrument [52,53]. As the CCI was originally developed for adult populations and has limited validation in children, values obtained in pediatric cases should be interpreted cautiously.
(g) Where available, echocardiographic findings;
(h) Outcomes: intubation (with/without extracorporeal membrane oxygenation, ECMO); cardiac arrest; and survival (with and without residual morbidity) vs. death.

### 2.6. Qualitative Assessment

Cases reported in the relevant studies were independently screened and rated according to Murad et al. [54] using four main domains (Selection, Ascertainment, Causality of case, and Reporting quality), comprising six dichotomous items. Each study was rated for each item as either low risk of bias (score +1) or high risk of bias (no score added), leading to a cumulative score ranging from 0 (high risk of bias) to 6 (low risk of bias; Appendix A, Table A3). Two investigators independently read the full-text versions of all included articles, rating their content in accordance with the aforementioned scoring system. Disagreements were resolved through discussion and consensus between the two reviewers or, when necessary, through consultation with the chief investigator (MR) as a third reviewer. In accordance with the original recommendations from Murad et al. [54], and consistently with the guidelines from the National Toxicology Program (NTP)’s Office of Health Assessment and Translation (OHAT; now the Health Assessment and Translation [HAT] group) [55,56], even studies with “high” or “unclear risk” ratings in one or more of the assessed domains were not excluded from the final body of evidence solely on this basis.

### 2.7. Data Analysis

Included case reports were separately pooled for RSV and hMPV and summarized using descriptive statistics. Continuous variables were reported as means with their corresponding 95% confidence intervals (95% CIs) and as median values with their interquartile ranges (IQRs), whereas categorical variables were reported as counts and percentages.

Univariate analyses were subsequently performed to compare the main continuous and categorical variables between hMPV and RSV cases. Categorical variables were compared using the chi-squared test with continuity correction for dichotomous variables, whereas continuous variables were compared using the Mann–Whitney U test.

Cases were then stratified according to age (<1 year, 1–17 years, and ≥18 years), and hMPV and RSV cases were compared within each age stratum. This analysis was undertaken to assess whether the differences observed between hMPV and RSV in the overall sample were consistent across age groups.

Multivariable analysis was subsequently performed using binary logistic regression, with hMPV versus RSV diagnosis as the dependent variable, to estimate the corresponding adjusted odds ratios (aORs). Accordingly, aOR values <1.00 indicated an association with RSV diagnosis, whereas aOR values >1.00 indicated an association with hMPV diagnosis. Explanatory variables were grouped into four a priori specified models, as follows:

- Model I included the main baseline characteristics of the patients: sex, age <18 years at hospital admission, and CCI.
- Model II focused on clinical presentation at hospital admission and included the variables entered in Model I together with fever, shortness of breath, dyspnea, chest pain, cough, sneezing, rhinorrhea, asthenia, convulsions, lethargy/altered mental status, any sign of arrhythmia, tachycardia, tachypnoea, hypotension, depressed ejection fraction (EF) on ultrasonography, and cardiac arrest.
- Model III focused on respiratory support and included the baseline variables from Model I together with high-flow oxygen therapy, continuous positive airway pressure (CPAP), mechanical ventilation, and extracorporeal membrane oxygenation (ECMO).
- Model IV focused on the main clinical outcomes and included the baseline variables from Model I together with diagnoses of myocarditis, pericarditis, rhythm abnormalities, heart failure, and myocardial infarction, as well as ICU admission and death.

Given the limited number of available cases, particularly in the hMPV group, these multivariable models were intended as exploratory analyses rather than confirmatory analyses. Accordingly, adjusted estimates were interpreted primarily in terms of their direction and uncertainty, with particular caution applied to associations characterized by wide confidence intervals.

Multiple correspondence analysis (MCA) was then performed as an exploratory analysis to assess multivariate patterns of clinical presentation. Clinical signs and symptoms were included as active categorical variables, whereas viral etiology (RSV vs. hMPV) was included as a supplementary qualitative variable and therefore did not contribute to the construction of the MCA dimensions. The first two dimensions were visualized, with individual cases grouped according to viral etiology and represented with 95% confidence ellipses.

### 2.8. Software

Screening of retrieved articles was performed using Mendeley Reference Manager (version 2.149.0; Mendeley Ltd.; New York, NY, USA). All calculations were performed using of R (version 4.6.0) [57] and RStudio (version 2026.08.2 Build 200; Posit Software, PBC; Boston, MA, USA), with the R packages “fmsb” (version 0.7.6), “epiR” (version 2.0.94), “dplyr” (version 1.2.1), “FactoMineR” (version 2.16), and “factoextra” (version 2.2.0). Plots were generated using the R packages “ggplot2” (version 4.0.2) and “ggpubr” (version 1.0.0), as well as GraphPad Prism version 11.0 (GraphPad Software LLC, Boston, MA, USA).

## 3. Results

### 3.1. Search and Selection Process

**Figure 1:**
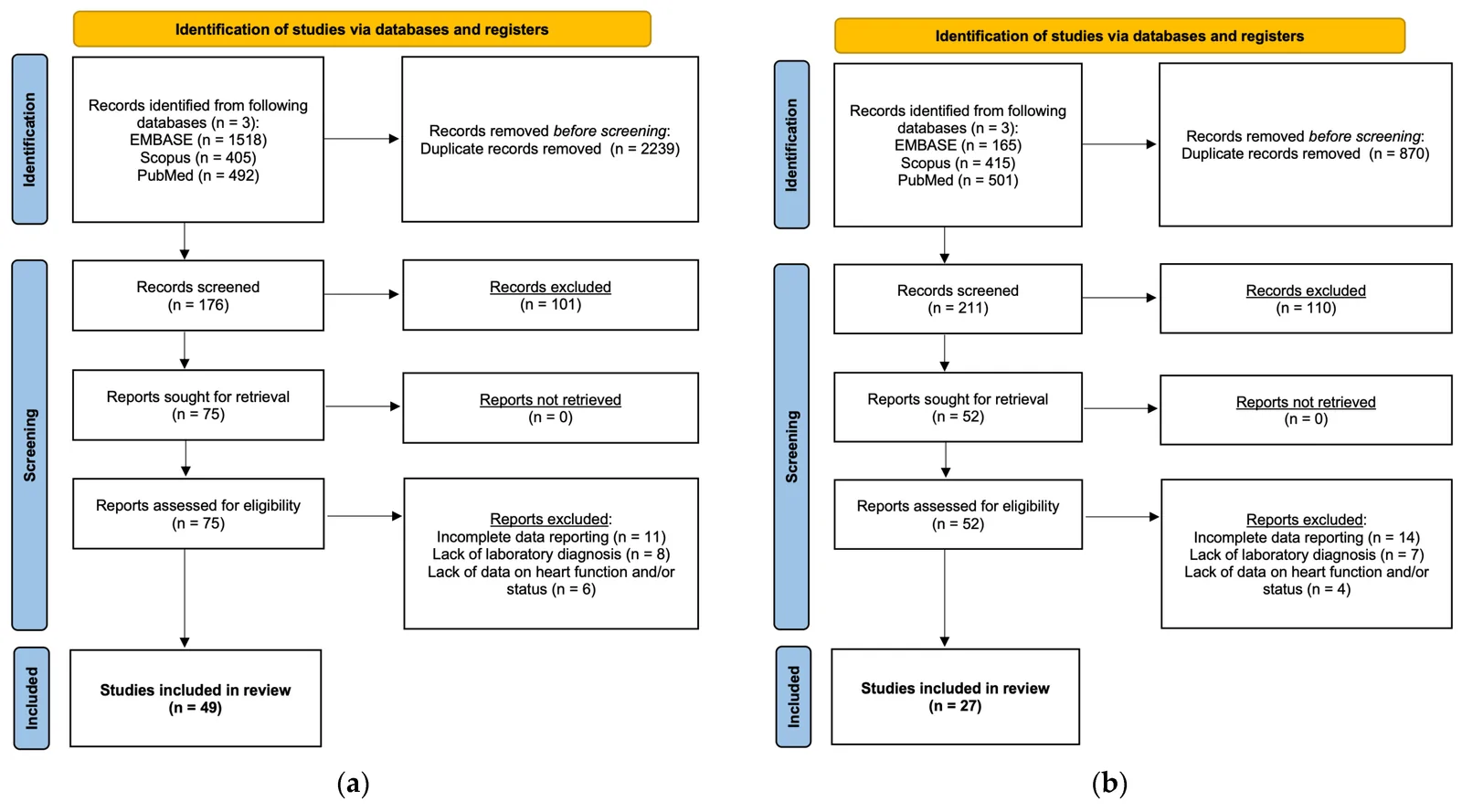
PRISMA flow chart of included studies on respiratory syncytial virus (**a**) and human metapneumovirus (**b**) cases with heart involvement [43,44].

As detailed in Table 1 and shown in Figure 1, a total of 3496 entries were identified across the queried databases, including 2415 records related to RSV (i.e., 1518 from Embase, 405 from Scopus, and 492 from PubMed), and 1081 related to hMPV (i.e., 165 from Embase, 415 from Scopus, and 501 from PubMed; Figure 1). After the removal of duplicates and screening of titles, abstracts, and full texts according to the eligibility criteria, a total of 49 papers on RSV (2.0% of the initial sample) [28,36,37,38,39,40,41,58,59,60,61,62,63,64,65,66,67,68,69,70,71,72,73,74,75,76,77,78,79,80,81,82,83,84,85,86,87,88,89,90,91,92,93,94,95,96,97,98,99] and 27 papers on hMPV infections (2.5% of the initial sample) [92,100,101,102,103,104,105,106,107,108,109,110,111,112,113,114,115,116,117,118,119,120,121,122,123,124,125] were included. Overall, 102 cases with cardiac complications were included in the analyses, of which 71 (69.6%) were associated with RSV infection and 31 (30.4%) with hMPV infection.

### 3.2. Characteristics of Retrieved Case Reports

Individual characteristics of retrieved cases are detailed in Appendix A, Table A2. Briefly, the included studies reported cases from 1973 to 2026 and were highly heterogeneous in terms of diagnostic approaches, testing strategies, and clinical follow-up. More than half of all cases (53.9%) were reported after the onset of the COVID-19 pandemic in 2020, and the proportion of hMPV cases reported during this period (83.9%) was significantly higher than that of RSV cases (40.8%; chi-squared test, *p* < 0.001).

**Figure 2:**
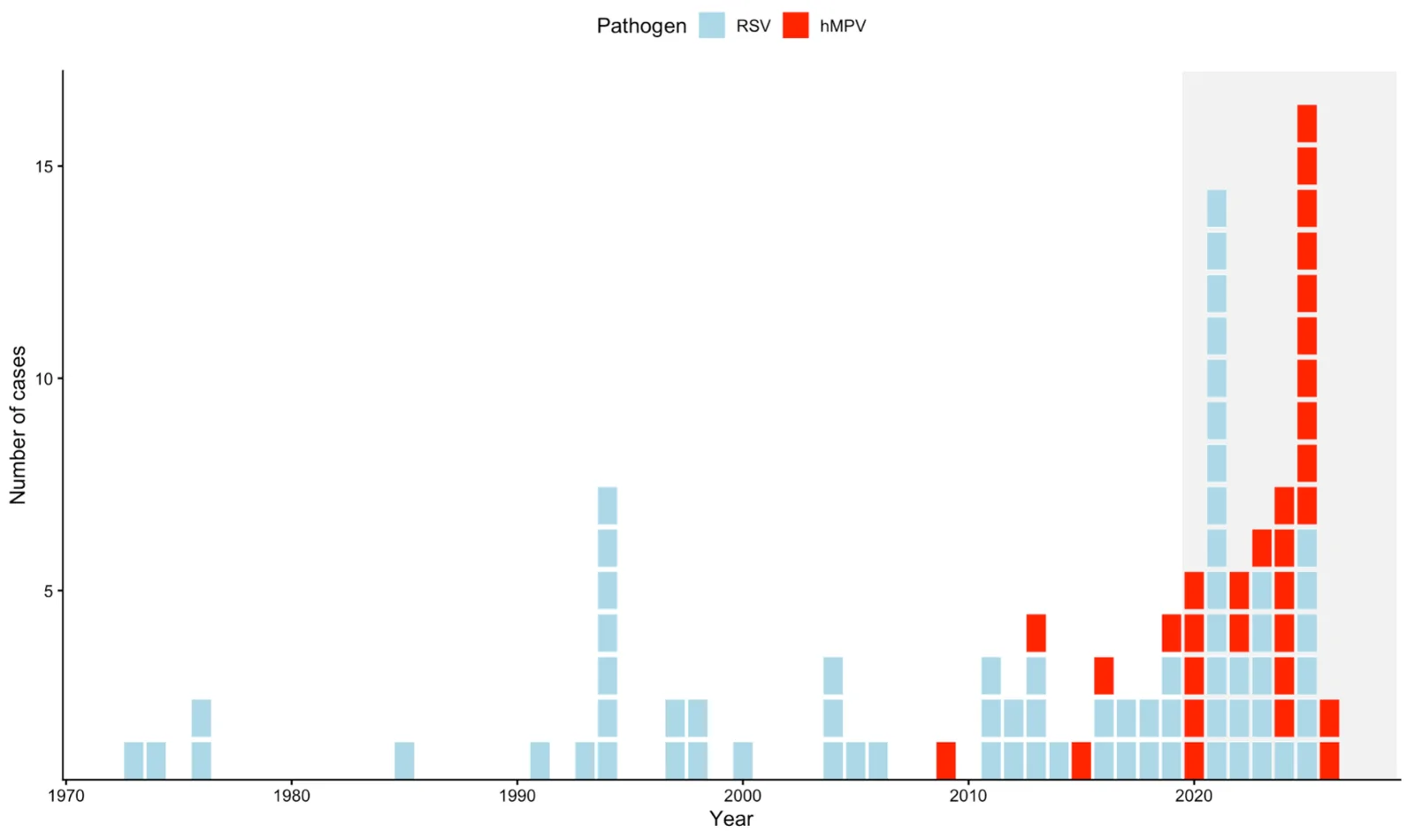
Distribution of retrieved case reports from a chronological point of view. Each square represents a single documented RSV or hMPV case. The gray area represents cases documented after the onset of the SARS-CoV-2 pandemic in 2020.

As shown in Figure 2, the chronological distribution of reported cases differed substantially between the two viruses. While cases of RSV with documented cardiac involvement have been reported since the early 1970s, hMPV-associated cases appeared only several decades later, reflecting the more recent identification of hMPV as a human pathogen.

Regarding the country of origin of the reports, most cases were reported from three countries, i.e., the USA (35.2% of RSV cases and 41.9% of hMPV cases), Japan (22.5% of RSV cases and 9.7% of hMPV cases), and Iran (5.6% of RSV cases and 12.9% of hMPV cases).

**Figure 3:**
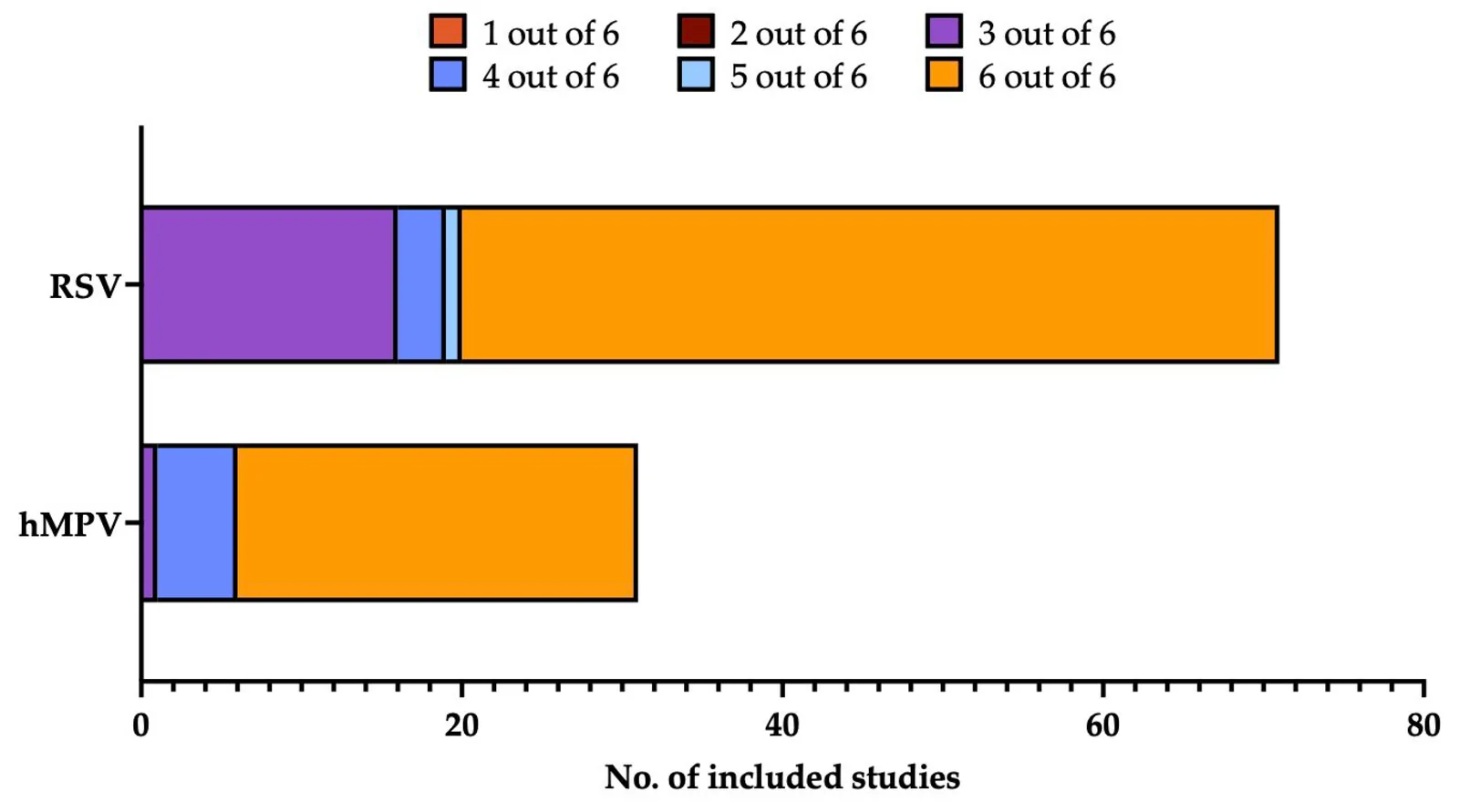
Summary of quality appraisal of included studies according to Murad et al. [54] for case reports and case series. A total of six domains were assessed by means of a dichotomous approach (i.e., low risk of bias vs. high risk of bias), leading to a sum score ranging from 0 to 6.

The overall quality of the reports is summarized in Figure 3 at the individual-case level.

Briefly, most reports achieved high appraisal scores according to the tool from Murad et al. [54], with 73.2% of RSV case reports and 80.6% of hMPV case reports achieving a quality score of five or six out of six. Although the body of evidence also included conference abstracts, no report received a score below three, whereas 26.7% of RSV case reports and 19.3% of hMPV case reports achieved scores of three to four (see Appendix A, Table A3 for individual scores).

### 3.3. Characteristics of Pooled Patients

#### 3.3.1. Individual Characteristics

**Figure A1:**
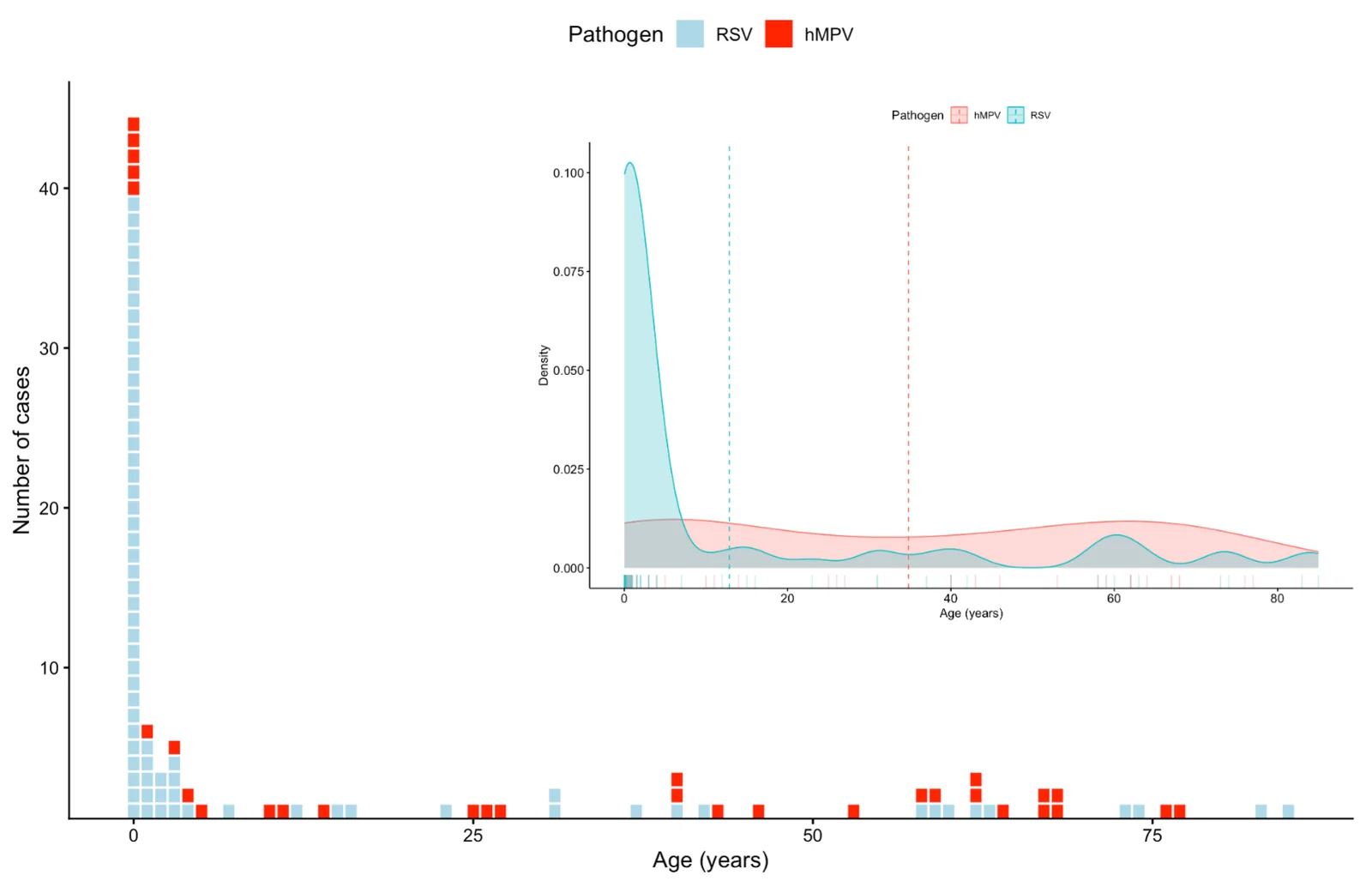
Distribution of documented cases of cardiac complications of infections due to respiratory syncytial virus (RSV) and human metapneumovirus (hMPV) by age at diagnosis. While the main figure reports each individual case and its cumulative raw number, the subfigure includes the cumulative density of reported cases. Scattered lines represent the median of RSV and hMPV cases.

Most hMPV and RSV cases were male (54.8% and 60.6%, respectively; *p* = 0.748) (Table 2). Patients with hMPV infection were significantly older than those with RSV infection, with a median age of 41.5 years (IQR 3.5 to 63.0) vs. 1.5 years (IQR 0.2 to 31.0). Consistent with this difference, the proportion of individuals aged less than 18 years was 38.7% among hMPV cases, compared with 74.6% among RSV cases (*p* < 0.001). More precisely, only 16.1% of hMPV cases were documented during the first year of life, whereas more than half of RSV cases involved infants aged < 1 year (57.7%), with 23.9% of RSV cases occurring within the first month of age (*p* < 0.001). The detailed age distribution is provided in Appendix A, Figure A1.

Most RSV cases (77.5%) had no reported coexisting comorbidity, compared with 41.9% of hMPV cases. Moreover, the proportion of cases with a moderate (CCI 3 to 4) or severe (CCI ≥ 5) comorbidity burden was significantly greater among hMPV than RSV cases (16.1% and 12.9% vs. 4.2% and 5.6%, respectively; *p* = 0.005). The detailed list of underlying comorbidities is provided in Appendix A, Table A4. Prematurity and a history of heart disease were numerically more frequent among RSV than hMPV cases (5.6% vs. 0, *p* = 0.427; and 12.7% vs. 0, *p* = 0.090), whereas chronic kidney disease was more frequent among hMPV cases (12.9% vs. 1.4%, *p* = 0.048).

**Figure 4:**
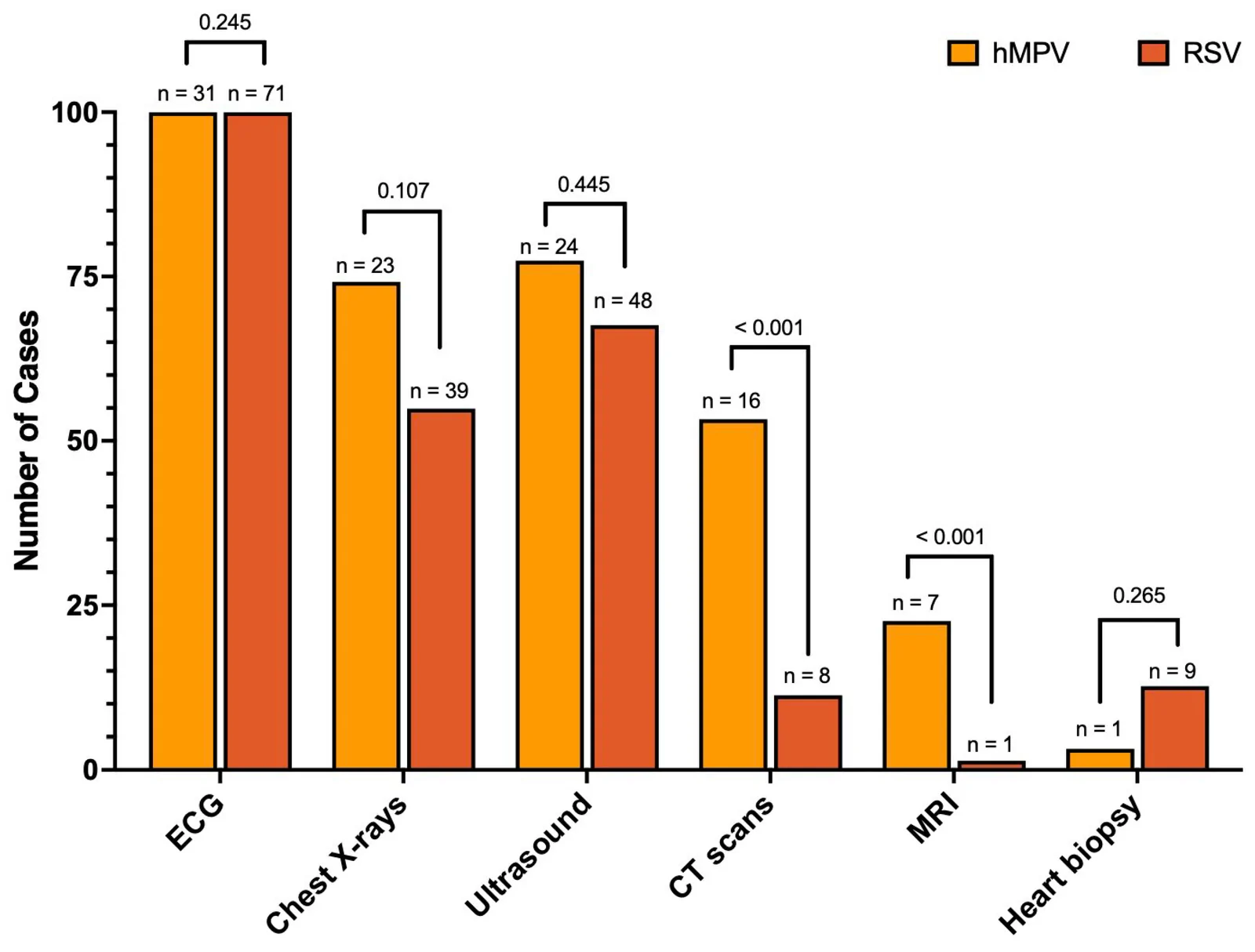
Summary of diagnostic work-up in sampled human metapneumovirus (hMPV) and respiratory syncytial virus (RSV). Note: ECG = electrocardiography; CT = computed tomography; MRI = magnetic resonance imaging.

Diagnostic imaging and instrumental investigations were not performed uniformly across RSV and hMPV cases (Figure 4). Ultrasonography, CXR, CT scans, and MR imaging were more frequently performed in hMPV cases (77.4%, 74.2%, 53.3%, and 22.6%, respectively) than in RSV cases (67.6%, 54.9%, 11.3%, and 1.4%, respectively). Differences in the use of CT and MRI were statistically significant (*p* < 0.001 for both comparisons).

#### 3.3.2. Main Characteristics at Hospital Admission

The time elapsed since symptom onset did not differ significantly between hMPV and RSV cases (6.1 days, 95% CI 3.6–8.7 vs. 4.9 days, 95% CI 3.4–6.5; *p* = 0.400). Overall, most patients presented with features consistent with LRTI (64.5% of hMPV cases and 67.6% of RSV cases). However, dyspnea (93.5% vs. 66.2%; *p* = 0.008) and chest pain (41.9% vs. 18.3%; *p* = 0.023) were significantly more frequent among hMPV than RSV cases. Shortness of breath (87.1% vs. 66.2%), cough (77.4% vs. 56.3%), fever at any time since symptom onset (67.7% vs. 56.3%), and asthenia (54.8% vs. 45.1%) were also more frequently reported among hMPV cases, although none of these differences reached statistical significance (all *p* > 0.05). Conversely, rhinorrhea was significantly more frequent among RSV cases (38.0% vs. 12.9%; *p* = 0.021), whereas the frequency of sneezing was comparable between RSV and hMPV cases (29.6% vs. 25.8%; *p* = 0.696). Approximately one-third of RSV cases presented with altered mental status, including lethargy, at hospital admission, compared with 16.1% of hMPV cases, although the difference was not statistically significant (*p* = 0.114). Signs of encephalopathy were reported in 9.7% of hMPV cases and 7.0% of RSV cases (*p* = 0.956).

The majority of both RSV (64.5%) and hMPV (77.9%) cases underwent ECG evaluation. Signs of arrhythmia were more frequently documented before hospital admission among RSV than hMPV cases (16.9% vs. 6.5%), although the difference was not statistically significant (*p* = 0.272). At hospital admission, the corresponding proportions were 56.3% and 29.0%, respectively, with arrhythmias being significantly more frequent among RSV cases (*p* = 0.011). Cardiac arrest before hospital admission or during the early phase of medical care was reported in 18.3% of RSV cases and 12.9% of hMPV cases, with no statistically significant difference between the two groups (*p* = 0.700).

Regarding heart rate characteristics (Table 3), tachycardia was the most frequently reported finding in both groups, occurring in 74.2% of hMPV cases and 53.5% of RSV cases. A normal heart rate was reported in 22.6% and 28.2% of hMPV and RSV cases, respectively. In six RSV cases (8.5%), heart rate could not be assessed because of ongoing cardiac arrest. Overall, the distribution of heart rate categories did not differ significantly between the two groups (*p* = 0.131). Blood pressure patterns were also similar between hMPV and RSV cases: normal blood pressure was reported in 61.3% and 62.0% of cases, respectively, while hypotension was reported in 29.0% and 28.2% of cases, respectively. Approximately half of hMPV cases had a normal respiratory rate (48.4%), compared with 57.7% of RSV cases, whereas tachypnoea was reported in 51.6% and 33.8% of hMPV and RSV cases, respectively. Finally, at hospital admission, body temperature was within the normal range in 61.3% of hMPV cases and 71.8% of RSV cases, whereas fever was reported in 35.5% and 21.1%, respectively. The overall distribution of body temperature categories did not differ significantly between the two groups (*p* = 0.087).

#### 3.3.3. Clinical Course and Outcome

Length of hospital stay was similar between the two groups (18.2 days, 95% CI 10.0–26.4 for hMPV vs. 14.8 days, 95% CI 11.7–17.9 for RSV; *p* = 0.988). Although the differences were not statistically significant, hMPV cases showed numerically higher frequencies of thrombotic and pulmonary complications during hospitalization. Deep vein thrombosis was reported in 9.7% of hMPV cases compared with 1.4% of RSV cases (*p* = 0.154), whereas diffuse alveolar hemorrhage occurred in 12.9% and 3.0% of hMPV and RSV cases, respectively (*p* = 0.147).

**Figure 5:**
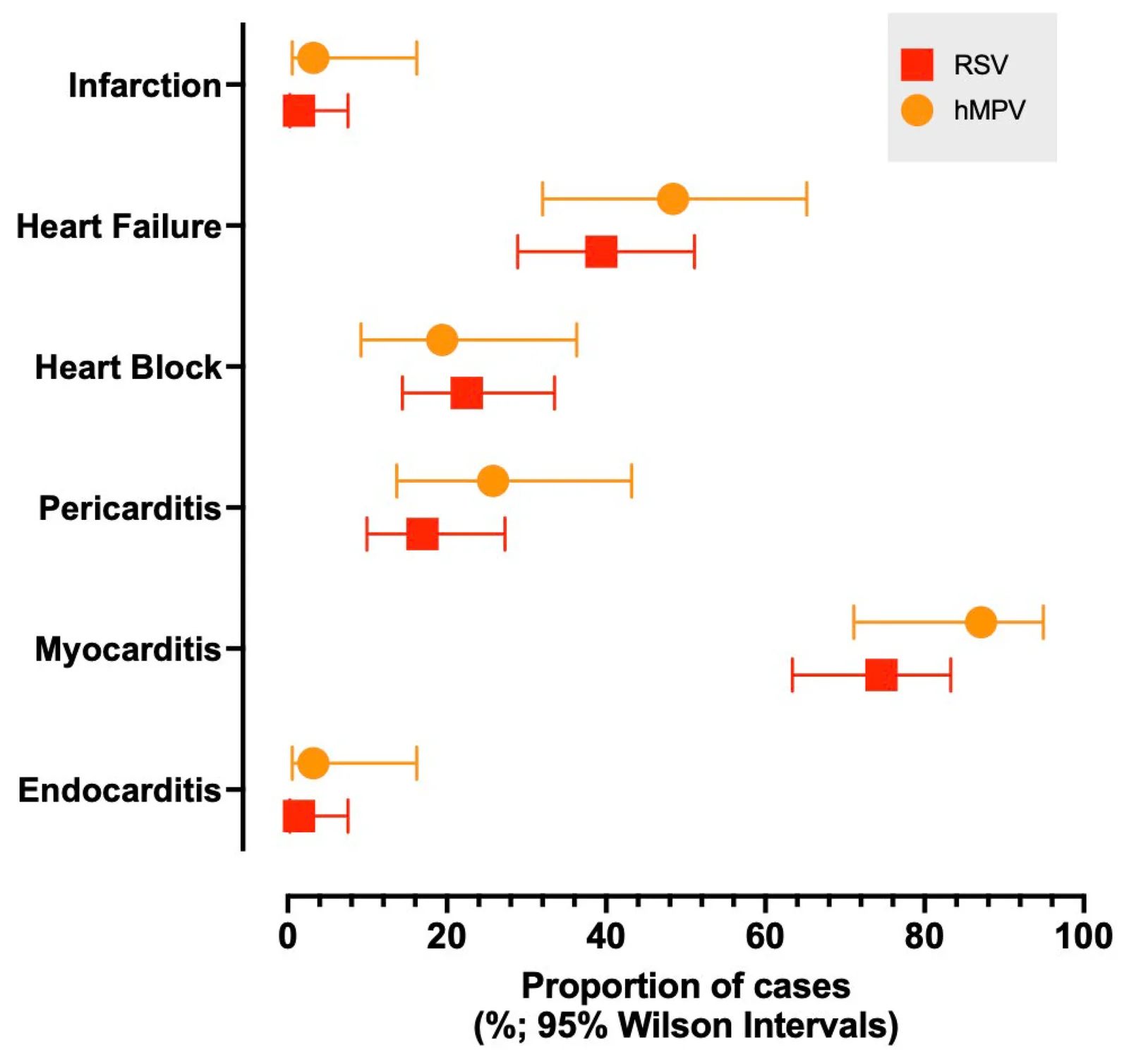
Cardiac manifestations of RSV and hMPV cases with their corresponding 95% Wilson Intervals.

The pattern of cardiac complications was broadly comparable between the two groups. Myocarditis was the most frequently reported diagnosis, occurring in 87.1% of hMPV cases (95% Wilson Interval [WI] 71.1 to 94.9) and 74.6% (95%WI 63.4 to 83.3) of RSV cases (*p* = 0.253), followed by signs of heart failure (48.4% [95%WI 32.0 to 65.2] vs. 39.4% [95%WI 28.9 to 51.1], respectively; *p* = 0.441) and pericarditis (25.8% [95%WI 13.7 to 43.2] vs. 16.9% [95%WI 9.9 to 27.3]; *p* = 0.253). One case of endocarditis was reported in each group (3.2% [95%WI 0.6 to 16.2] vs. 1.4% [95%WI 0.2 to 7.6]; *p* = 1.000). Signs of heart block were observed in 19.4% of hMPV cases (95%WI 9.2 to 26.3) and 22.5% of RSV cases (95%WI 14.4 to 33.5; *p* = 0.922). Only two cases of myocardial infarction were documented, one in each group (3.2%, 95%WI 0.6 to 16.2 vs. 1.4%, 95%WI 0.2 to 7.6; *p* = 1.000) (Figure 5).

Coinfections were significantly more frequent among hMPV than RSV cases (25.0% vs. 1.4%; *p* < 0.001). Most coinfections involved bacterial pathogens (19.4% vs. 1.4%, respectively). The most frequently reported bacterial pathogen was *Streptococcus pneumoniae*, which was identified exclusively among hMPV cases (9.7%).

**Figure A2:**
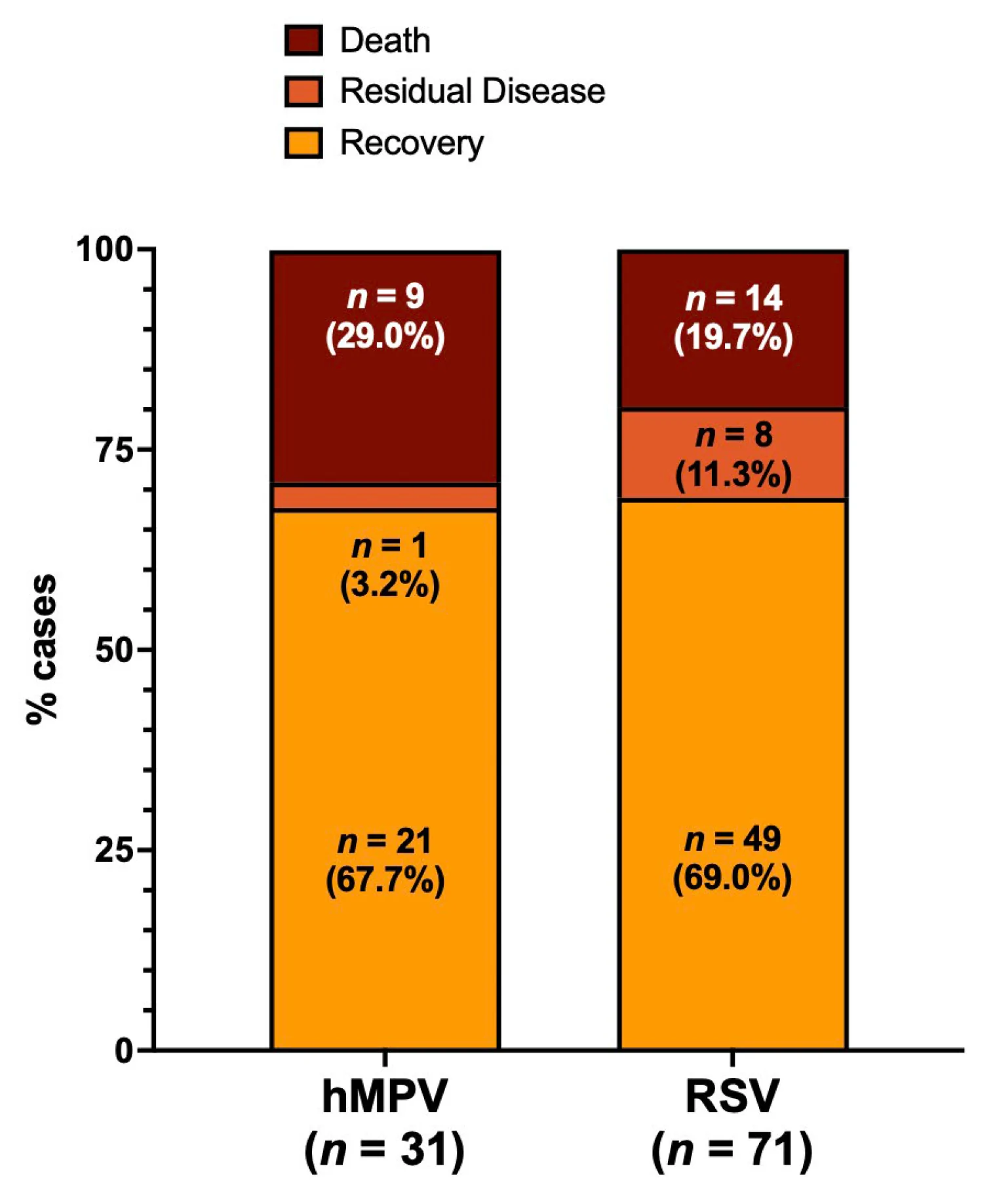
Case-level outcomes reported in the present review.

**Figure A3:**
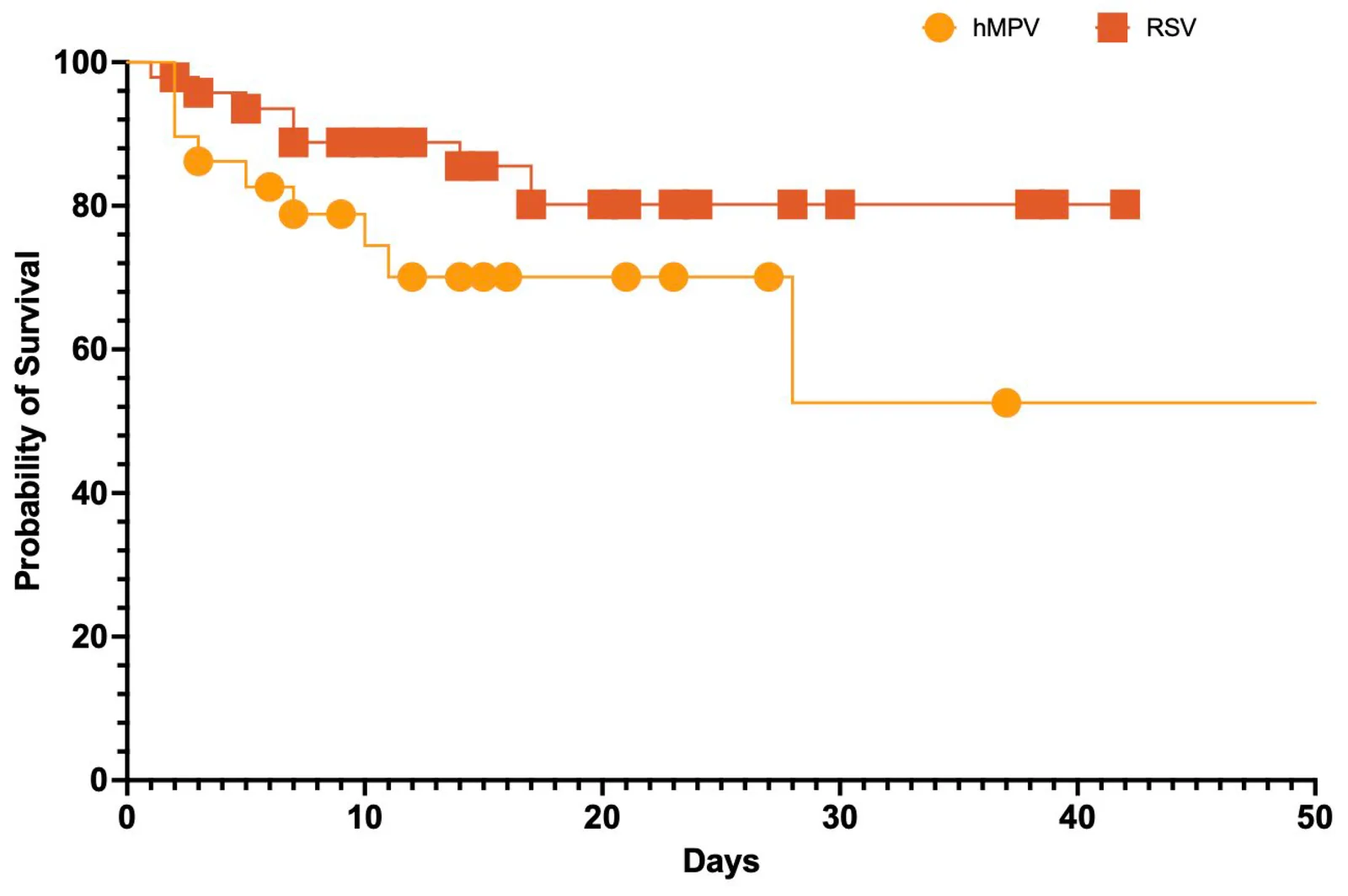
Analysis of proportional survival of respiratory syncytial virus (RSV) cases and human metapneumovirus (hMPV) cases by means of Kaplan–Meier statistics. No significant differences were identified at the Log-Rank Mantel–Cox statistic (chi-squared test 2.105; *p*-value = 0.147).

Regarding clinical outcomes, ICU admission was significantly more frequent among hMPV than among RSV cases (96.8% vs. 77.5%; *p* = 0.034). Overall, 23 deaths were reported, nine among hMPV cases and 14 among RSV cases (29.0% vs. 19.7%, respectively). Residual disease, primarily consisting of persistent arrhythmias, was documented in eight RSV cases (11.3%) compared with one hMPV case (3.2%), whereas complete recovery of cardiac function was reported in 67.7% of hMPV cases and 69.0% of RSV cases (Appendix A, Figure A2). The overall distribution of outcomes did not differ significantly (*p* = 0.299). Similarly, Kaplan–Meier survival analysis showed no statistically significant difference between the two groups (estimated mean survival: 34.9 days, 95% CI 30.3 to 39.4 for RSV vs. 54.9 days, 95% CI 31.8 to 75.9 for hMPV; Mantel–Cox log-rank chi-squared test = 2.105; *p* = 0.147; Appendix A, Figure A3).

#### 3.3.4. Electrocardiographic Features

Available ECG findings are summarized in Table 4. Overall, electrocardiographic patterns were largely comparable between hMPV and RSV cases.

Patients with hMPV infection more frequently had sinus rhythm than those with RSV infection (35.5% vs. 26.8%), although the difference did not reach statistical significance (*p* = 0.374). The overall distribution of tachyarrhythmias was similar between the two groups (*p* = 0.181), with sinus tachycardia and supraventricular tachycardia representing the most common findings. Ventricular tachycardia was reported in only one RSV case. Similarly, bradyarrhythmias occurred at comparable frequencies in the two groups. Conduction disturbances were numerically more frequent among RSV cases, primarily due to a higher incidence of atrioventricular block. Finally, ventricular repolarization abnormalities were common in both groups, with ST-segment changes representing the predominant electrocardiographic finding (32.3% vs. 34.3%; *p* = 1.000). T-wave inversion (6.5% vs. 14.3%; *p* = 0.430) and QRS abnormalities (12.9% vs. 14.3%; *p* = 1.000) were also reported at comparable frequencies in the two groups.

#### 3.3.5. Radiological and Imaging Features

Findings from CXR, CT scans, and cardiac ultrasonography have been reported in Table 5 and were largely comparable between hMPV and RSV cases.

On CXR, hMPV and RSV cases showed comparable frequencies of cardiomegaly (30.4% vs. 48.7%; *p* = 0.253), pulmonary edema (30.4% vs. 38.5%; *p* = 0.716), pulmonary opacities (56.5% vs. 48.7%; *p* = 0.741), and infiltrative lesions (47.8% vs. 61.5%; *p* = 0.431). Infiltrative lesions were also frequently identified on chest CT, occurring in 73.3% of hMPV cases and 62.5% of RSV cases (*p* = 0.951). Pleural effusions were documented in 50.0% of hMPV cases and 75.0% of RSV cases undergoing chest CT, with no statistically significant difference between the groups (*p* = 0.464). Cardiac lesions were documented on chest CT in approximately one-quarter of cases in both groups (23.5% in hMPV vs. 25.0% in RSV; *p* = 1.000). MRI was performed in only eight cases, seven of which involved hMPV infection. Among these seven hMPV cases, six showed evidence of cardiac lesions.

Cardiac ultrasonography provided both anatomical and functional information. The most frequently reported finding was evidence of myocarditis, with similar frequencies among hMPV and RSV cases (83.3% vs. 85.4%; *p* = 1.000), followed by signs of pericarditis (41.7% vs. 39.6%; *p* = 1.000). Endocarditis was documented in only two cases, one in each group (*p* = 1.000). Findings consistent with Takotsubo syndrome were reported exclusively among hMPV cases (12.5%), although the between-group difference did not reach statistical significance (*p* = 0.061). Regarding left ventricular systolic function, ejection fraction was normal in 25.0% of hMPV cases and 29.2% of RSV cases, moderately depressed (30–50%) in 33.3% and 43.8%, respectively, and severely depressed (<30%) in 41.7% and 27.1%, respectively. Overall, the distribution of ejection fraction categories did not differ significantly between the two groups (*p* = 0.450).

#### 3.3.6. Histopathological Findings

Histopathological findings were available for only nine RSV cases and one hMPV case, and the corresponding results are summarized in Table 6. Briefly, RSV was identified in cardiac tissue in only two cases [41,70]. Myocarditis was the most frequently reported histopathological finding, documented in six of the nine RSV cases, with lymphocytic infiltration reported in three of these six cases.

### 3.4. Age-Stratified Analysis

In age-stratified analyses (Table 7), differences between hMPV and RSV cases were generally attenuated within individual age groups, which may partly reflect the small numbers of cases within individual strata. Nevertheless, some differences observed in the overall analysis remained apparent within specific age strata, although most comparisons did not reach statistical significance.

### 3.5. Multivariable Analysis

The results of the multivariable analyses are reported in Appendix A, Table A5. In Model I, which included baseline patient characteristics, no statistically significant associations with viral diagnosis were identified. In Model II, which additionally accounted for clinical features at hospital admission, dyspnea (aOR 16.280, 95% CI 1.370–193.475) and cough (aOR 6.059, 95% CI 1.062–34.552) were associated with higher odds of hMPV rather than RSV diagnosis. Conversely, rhinorrhea (aOR 0.031, 95% CI 0.003–0.286) and lethargy and/or altered mental status (aOR 0.103, 95% CI 0.015–0.712) were associated with lower odds of hMPV rather than RSV diagnosis, and were therefore more strongly associated with RSV diagnosis. No statistically significant associations were identified in Model III, which focused on respiratory support requirements. In Model IV, which focused on the main clinical outcomes, ICU admission was associated with higher odds of hMPV rather than RSV diagnosis (aOR 11.033, 95% CI 1.125–108.173).

Given the limited sample size and the wide confidence intervals of several estimates, these findings should be regarded as exploratory and interpreted cautiously, as sparse-data effects and model instability cannot be excluded.

### 3.6. Multiple Correspondence Analysis

**Figure 6:**
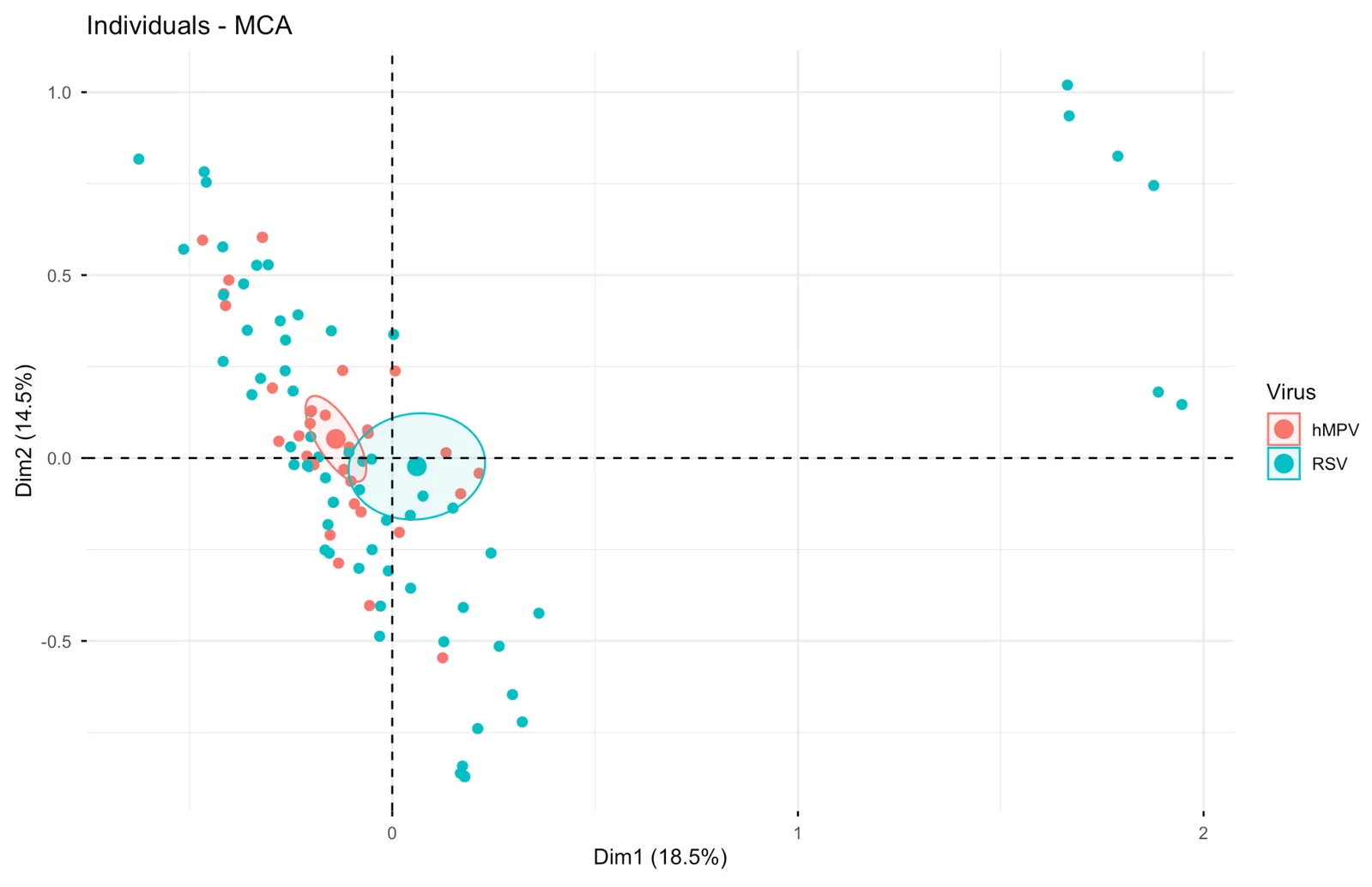
Multiple correspondence analysis (MCA) of clinical presentation in patients with respiratory syncytial virus (RSV) and human metapneumovirus (hMPV) infection. Individual patients are represented according to the first two MCA dimensions and grouped by viral etiology (RSV or hMPV). Ellipses represent the 95% confidence regions around each group. Viral etiology was included as a supplementary qualitative variable and therefore did not contribute to the construction of the MCA dimensions.

**Figure A4:**
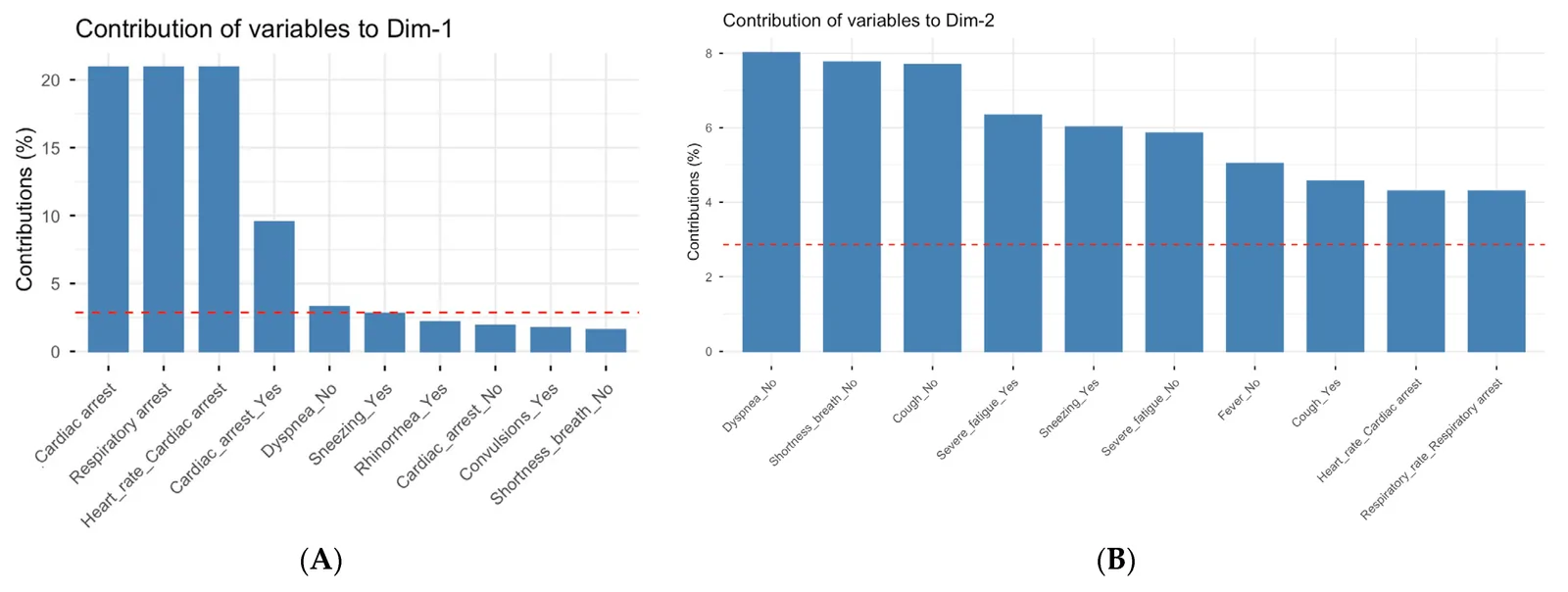
Contributions of clinical variable categories to the first two dimensions of the multiple correspondence analysis (MCA). Panels (**A**,**B**) show the ten variable categories with the largest contributions to Dimensions 1 and 2, respectively. The dashed red line represents the expected average contribution under equal contribution of all categories.

Multiple correspondence analysis (MCA) was performed to explore overall patterns of clinical presentation according to viral etiology. The first two dimensions accounted for 18.5% and 14.5% of the total inertia, respectively (Appendix A, Figure A4A,B), for a cumulative 33.1%. When RSV and hMPV were projected as supplementary categories, the two groups showed substantial overlap in the MCA space, with no clear separation in overall clinical presentation according to viral etiology (Figure 6). Consistently, neither viral etiology was significantly associated with either of the first two dimensions, based on the corresponding v-test values (all |v| < 1.96).

Examination of category contributions provided further insight into the structure of the two dimensions. Dimension 1 (Dim-1) was predominantly driven by categories related to cardiac and respiratory arrest and heart rate, whose contributions were markedly higher than those of the remaining clinical features. Dim-1 therefore appeared to primarily capture patterns associated with the most severe acute clinical presentations. In contrast, contributions to Dimension 2 (Dim-2) were more evenly distributed across multiple clinical features. The largest contributions were associated with the absence of dyspnea, shortness of breath, and cough, followed by severe fatigue, sneezing, fever, and the presence of cough. Dim-2 therefore appeared to reflect heterogeneity in respiratory and systemic clinical presentation rather than being dominated by a single clinical feature.

## 4. Discussion

### 4.1. Summary of Main Results

In the present systematic review, we retrieved 102 case reports describing cardiac complications associated with RSV or hMPV infection. Of these, 71 (69.6%) were associated with RSV infection, while the remaining 31 (30.4%) were associated with hMPV. Most cases were reported after the onset of the COVID-19 pandemic in 2020 (53.9%), with a significantly higher proportion of hMPV cases (83.9%) than RSV cases (40.8%), possibly reflecting the greater availability of testing options for suspected respiratory pathogens. Although hMPV-associated cases were significantly older (median 41.5 years, IQR 3.5 to 63.0) than RSV cases (median 1.5 years, IQR 0.2 to 31.0), RSV cases were highly clustered within the first year of life (57.7%), with a substantial proportion occurring among infants aged <1 month (23.9%). Moreover, hMPV cases had a higher burden of comorbidities, with a CCI >2 reported in 29.0% of hMPV cases compared with 9.8% of RSV cases. Despite these differences in baseline characteristics, most clinical features were broadly comparable between the two groups, as further supported by the MCA. Indeed, RSV and hMPV cases showed substantial overlap in the MCA space, indicating no clear separation in their overall clinical presentation by viral etiology. In multivariable analyses, the main clinical features associated with hMPV etiology included dyspnea (aOR 16.280, 95% CI 1.370 to 193.475) and cough (aOR 6.059, 95% CI 1.062 to 34.552), while rhinorrhea (aOR 0.031, 95% CI 0.003 to 0.286), and lethargy/altered mental status (aOR 0.103, 95% CI 0.015 to 0.712) were associated with RSV rather than hMPV diagnosis. ICU admission was also associated with hMPV etiology (aOR 11.033, 95% CI 1.125–108.173). These differences should nevertheless be interpreted in light of the marked age heterogeneity of the included cases, which may have influenced both the reporting of symptoms and complaints and the underlying burden of comorbidities and frailty, including age-related conditions such as diabetes and chronic kidney disease.

### 4.2. Generalizability

Although neither RSV nor hMPV has been shown to exhibit specific cardiac tropism, both pathogens have been associated with adverse cardiovascular outcomes [27,28]. Cardiovascular complications have been reported among patients with both RSV and hMPV infection [29,30,31]. For example, Ivey et al. [29] reported that hospitalizations due to RSV-associated respiratory illness can be complicated by cardiovascular events in 14% to 22% of adult patients. Their results were based on seven studies published in the previous two decades and included 1134 patients [34,48,122,123,124], most of whom were adults or older adults. In most cases, RSV was associated with worsening of underlying medical conditions, such as chronic heart failure, but a substantial proportion of cases also exhibited newly diagnosed conditions such as arrhythmias and acute coronary and/or myocardial events [34,126,127,128]. Recent studies have consistently suggested an association between RSV infection and an increased risk of cardiovascular events and exacerbation of pre-existing cardiovascular conditions, particularly among individuals with documented comorbidities [129,130,131]. For example, in the retrospective study by Sudnik et al. [131] including a total of 471 patients, 25% developed either newly diagnosed heart failure or worsening of pre-existing heart failure, with atrial fibrillation reported in 13% and myocardial infarction in 9%. Similarly, the matched pair study of Hviid et al. [130] documented a 4.7% risk difference (95% CI 4.0 to 5.4) risk difference for cardiovascular events among individuals with a previous diagnosis of RSV infection, particularly among older individuals (+7.9%, 95% CI 5.4 to 10.5), those with diabetes (+7.5%, 95% CI 4.5 to 10.5), and those with pre-existing cardiovascular disease (+12.0%, 95% CI 8.8 to 15.1). Moreover, a more recent systematic review and meta-analysis by Montiel et al. [132] suggested that among hospitalized patients with documented RSV infection, up to 19.2% (95% CI 15.2 to 24.2) experienced a cardiac event, 15.7% (95% CI 14.8 to 16.5) experienced newly diagnosed or worsening heart failure, while 5.4% (95% CI 3.1 to 9.5) developed an acute coronary syndrome. Where direct comparisons with influenza infection were available, RSV infection was associated with a higher risk of cardiac complications (RR 1.2, 95% CI 1.1 to 1.4), particularly worsening of heart failure (RR 1.3, 95% CI 1.1 to 1.6). Although chronic heart disease has been historically considered a high-risk comorbidity for RSV infection [4,133,134,135,136], evidence regarding cardiac complications in pediatric RSV cases is more limited [79], particularly for myocarditis and newly diagnosed arrhythmias. While substantial evidence links newly diagnosed pediatric myocarditis to respiratory pathogens such as SARS-CoV-2 [137], influenza virus [138,139], as well as to enterovirus and parvovirus B19 [137], much of the available evidence specifically addressing RSV-associated myocarditis and arrhythmias derives from case reports and case series, which are summarized in the present review.

Evidence regarding hMPV-associated cardiovascular complications is even more limited and less well established. In a recent systematic review by Loubet et al. [140] including a total of nine studies, a very high prevalence of pre-existing cardiovascular conditions was identified (median: 40.2%, range: 10.3% to 80%), while the incidence of newly diagnosed conditions ranged from 3.5% to 22% because of heterogeneous case definitions and reported outcomes. A recent report from Jubair et al. [141] also suggested that hMPV cases may experience a 10-fold increase in the incidence of cardiac events, including newly diagnosed arrhythmias (33%) and myocarditis (10%). Similar to RSV, data on pediatric patients are less well defined. According to a retrospective study by Leister et al. [142], hMPV infection was identified in 3.1% of children admitted to the pediatric ICU of the University Hospital of Cologne between March 2018 and April 2020. In this small but detailed report, most patients had no underlying conditions. However, the limited available evidence does not allow firm conclusions regarding the role of pre-existing risk factors in the development of hMPV-associated cardiovascular complications.

By summarizing available data on the clinical features of cardiovascular complications associated with RSV and hMPV infections, our study may contribute to a better understanding of this topic. However, the generalizability of the present findings requires careful consideration for several reasons.

First, the included cases represent a highly heterogeneous population in terms of age, comorbidity burden, geographical origin, diagnostic work-up, and historical period. In particular, RSV cases were retrieved over a broader timeframe, ranging from the early 1970s to 2026, while hMPV cases were clustered in more recent years; moreover, their demographic features differed substantially. More precisely, RSV cases were substantially younger than hMPV cases, and a very large proportion of RSV-associated cardiac complications were reported during infancy. These results were not unexpected, as RSV in the general pediatric population usually affects younger individuals than hMPV [136,143,144,145], while their occurrence among older individuals is more comparable [146,147,148,149]. In fact, age-stratified sensitivity analyses generally attenuated the differences observed in the overall sample, suggesting that at least part of the apparent heterogeneity between RSV and hMPV may reflect differences in the populations in which cardiac complications have been reported rather than pathogen-specific phenotypes. Accordingly, the present findings should primarily be regarded as a characterization of the spectrum of reported cardiac involvement associated with these infections rather than as estimates of its frequency or relative risk in the general population.

Second, the pooled case-based evidence cannot determine whether cardiovascular complications are directly caused by RSV or hMPV or arise indirectly through systemic mechanisms. Direct viral involvement of myocardial or pericardial tissue has been documented in individual reports [35,36,37,38,39,40], but such evidence remains uncommon, and only a limited number of reported cases benefited from specific sampling [28,41,62,66,70,79,95,121]. Although most cases reported diagnoses of myocarditis and/or pericarditis, histopathological findings were largely nonspecific, including lymphocytic infiltration compatible with viral myocarditis, while the pathogen was directly identified in cardiac tissue in only two cases, both involving RSV [41,70]. In other words, although direct myocardial or pericardial viral involvement may occur [41], and even rhythm disturbances have been reported (including supraventricular tachycardia, multifocal atrial tachycardia and atrial flutter, ventricular tachycardia, atrioventricular block, and even ventricular fibrillation) [42], it remains unclear whether these conditions result from direct tissue infection or from systemic inflammatory, hypoxic, ischemic, or hemodynamic mechanisms associated with viral infection [27]. As recently stressed by Sozzi et al. [150], a viral pathogen can cause arrhythmias through several molecular and immunopathogenic mechanisms, including direct pathogen-mediated cytolysis with increased release of pro-inflammatory mediators; myocardial edema with subsequent cytokine release and cell death; direct effects on gap junctions through altered connexin expression; acute ischemia, microvascular dysfunction, and vasospasm; disturbances in electrolyte metabolism, particularly intracellular calcium homeostasis; ion-channel dysfunction; unmasking of underlying cardiomyopathy or other conditions predisposing to cardiomyopathy; persistent chronic inflammation with myocardial scar formation; prolonged ventricular dysfunction; and electrical remodeling. A similar uncertainty has been recently highlighted regarding neurological complications of RSV infections [16]: although RSV has been occasionally identified in samples from patients with central nervous system complications during or shortly after a documented episode of RSV-associated LRTI, many cases of RSV-associated encephalitis/encephalopathy may instead be related to severe respiratory disease and consequent hypoxia.

### 4.3. Implications for Clinical Practice and Current and Future Preventive Perspectives

A key message emerging from our findings is that RSV and hMPV show substantial overlap not only in their general clinical presentation but also in their reported cardiac manifestations. Myocarditis represented the predominant cardiac manifestation for both pathogens (87.1% for hMPV vs. 74.6% for RSV), and the reported spectrum of cardiac complications included rhythm and conduction disturbances, heart failure, pericardial involvement, and, more rarely, other cardiac complications. These manifestations are also shared with cardiovascular syndromes associated with other respiratory viruses [137,138,150], but the substantial overlap between RSV and hMPV observed in the MCA suggests that viral etiology alone is unlikely to provide sufficient information for anticipating the clinical phenotype of cardiac involvement. Although ICU admission was more frequent among reported hMPV cases, requirements for respiratory support and clinical outcomes did not differ substantially according to viral etiology. Moreover, as previously noted, the age-stratified analysis suggested that the higher occurrence of ICU admissions among hMPV cases may partly reflect differences in age and comorbidity burden rather than pathogen-specific characteristics.

As a consequence, rather than supporting systematic cardiac screening of all infected patients, these findings may justify a low threshold for cardiac assessment in patients presenting with severe disease, hemodynamic instability, chest pain, unexplained tachycardia or arrhythmias, or other findings suggestive of myocardial involvement [127,128,129,130,131,140]. In such patients, ECG and echocardiography may be reasonable first-line investigations [146,147,148,149], with more advanced imaging considered according to clinical findings and the overall clinical setting [151,152,153,154].

Given the potential severity of cardiac complications associated with RSV and hMPV infections, including the potential loss of functional independence after hospital discharge [30,127,131,155,156], available data underscore the clinical and public health value of preventive strategies. Although several monoclonal antibodies targeting hMPV are currently under investigation [157,158], commercially available options are currently limited to RSV infection and include passive immunization of infants with monoclonal antibodies (mAbs), maternal vaccination, and vaccination of older adults.

The first mAb commercially available for RSV prevention was palivizumab. Despite its documented effectiveness, its use was largely restricted to high-risk pediatric populations [159,160,161,162] because of its substantial direct and indirect costs [162,163]*,* which, in turn, were due to its relatively short duration of protection and the resulting need for monthly, weight-dependent doses (i.e., 15 mg/kg) [160,164,165]. Therefore, universal immunization campaigns against RSV in newborns have become feasible only after the licensing of nirsevimab (commercial name: Beyfortus, AstraZeneca [Södertälje, Sweden], and Sanofi [Gentilly, France]), a long-acting monoclonal antibody directed against site ø of the prefusion F protein [4,166,167]. Since its introduction, the effectiveness of nirsevimab in preventing severe LRTI [168,169,170] has been documented in several real-world studies [168,169,170,171,172,173,174,175], with early estimates of immunization effectiveness of approximately 90.5% (95% CI 87.1 to 93.0), and a low frequency of reported adverse reactions and side effects [144]. Subsequent observational studies have broadly supported these initial findings [135]. Clesrovimab (formerly MK-1654, marketed as Enflonsia™) is a fully human IgG1 monoclonal antibody developed by Merck & Co. (Rahway, NJ, USA) that targets antigenic site IV of the RSV fusion (F) glycoprotein, a distinct and non-overlapping epitope compared with site Ø targeted by nirsevimab [176,177,178]. Recently licensed in North America and the European Union, phase 2b/3 studies documented 60.4% (95% CI, 44.1–71.9; *p* < 0.001) immunization efficacy against RSV-associated medically attended lower respiratory infection (MALRI), and against RSV-associated hospitalizations (immunization efficacy 84.2%, 95% CI, 66.6–92.6). Moreover, the adult RSVpreF vaccine can also be administered during pregnancy to provide passive protection to the infant. As a substantial proportion of documented RSV complications in our sample occurred among infants aged <30 days, maternal vaccination may represent a particularly relevant preventive strategy for this age group. Available studies have reported crude vaccine effectiveness (VE) during the first three months of age ranging from 57.3% (95% CI 23.6 to 75.9) [179] to 84.6% (78.7 to 88.8) [180], compared with a range of 70% (95% CI 28 to 88) [179] to 82.9% (95% CI 75.9 to 87.8) [180] for adjusted VE estimates. At six months of age, crude VE ranged from 68.2% (95% CI 33.1 to 84.9) [181] to 84.1% (95% CI 76.7 to 89.0) [182], with corresponding adjusted estimates ranging from 66.1% (95% CI 30.1; 83.8) [183] to 78.7% (95% CI 51.4 to 90.8) [181].

Older adults may also benefit from RSV vaccination. Three vaccines have been authorized for clinical practice in the USA and/or EU [184,185]: RSVpreF, RSVpreF3, and mRNA-1345. The efficacy of RSVpreF, RSVpreF3 and mRNA-1345 in preventing RSV-associated disease and hospitalization has been evaluated in several RCTs, whose findings was reviewed by German Standing Committee on Vaccination (STIKO) [186], Riccò et al. [187], and Advisory Committee on Immunization Practices of the United States [188] around the time of their commercialization, and subsequently complemented by evidence from observational studies [189,190,191], including studies from Tartof et al. [192] (VE 91%, 95% CI 25 to 99), Symes et al. [193] (VE 82%, 95% CI 25 to 99); Bajema et al. [194] (VE 81%, 95% CI 69 to 88), and Surie et al. [195] (VE 77%, 95% CI 73 to 79).

Briefly, these vaccines have shown favorable safety profiles and effectiveness against RSV-associated disease and its complications, although specific focus on preventing cardiac complications remains more limited [196,197]. Early observational evidence suggests that RSV vaccination may reduce the risk of cardiovascular complications [196], while its effectiveness against cardiac arrhythmias remains more uncertain. Future development of vaccines against hMPV, as well as combined RSV-hMPV vaccines, may further expand preventive options against the burden associated with these viruses.

### 4.4. Limitations and Implications for Future Studies

Despite their potential relevance to clinical and epidemiological practice, our results are affected by substantial limitations stemming from the nature of the source studies and the available data.

First of all, although most reports achieved relatively high appraisal scores, the evidence included in this review consisted primarily of case reports and case series, and the total number of pooled subjects remains low, particularly when compared with the estimated global burden of incident cases of RSV- and hMPV-associated LRTI. In this regard, with most cases clustered in a limited number of countries, the sampled cases may partly reflect differences in healthcare resources, diagnostic capacity, and research activity across reporting centers. As a high proportion of global cases of both RSV and hMPV occur in resource-limited settings [2,10,11,13,14,17,24], our 102 cases may in fact represent the “tip of the iceberg” of cardiac complications associated with both pathogens, with limited representativeness and a substantial risk of preferential reporting of more severe, unusual, or clinically noteworthy cases. This is a common caveat when dealing with case reports and case series, which are primarily descriptive designs used to report novel, unusual, or atypical features, and substantial debate remains regarding their role in evidence synthesis [54,198]. Consequently, reviews pooling individual data from case studies and case reports may be particularly susceptible to reporting and selection biases [199,200].

Second, because of the case-based nature of the available evidence, data were generally collected retrospectively and were often incomplete or inconsistently reported [200]. The heterogeneous availability of clinical and outcome data may reflect not only differences in diagnostic resources but also differences in data collection and reporting across centers. This issue may be particularly relevant to case series spanning extended periods such as the ones by Donnerstein et al. [65], Kawashima et al. [79], Ramzali et al. [92], as well as Sakha et al. [36], which may have been affected by changes in diagnostic options made available over time, and by inter-observer inconsistencies. This limitation is particularly relevant to imaging studies, which were not systematically performed and were obtained using heterogeneous CT and MR imaging technologies. In particular, the higher use of CT and MRI among hMPV cases may have increased the likelihood of detecting cardiac abnormalities, potentially contributing to an apparent greater severity of hMPV-associated cases compared with RSV-associated cases. Moreover, because the decision to perform advanced imaging may itself have been influenced by clinical suspicion of greater disease severity, selection and ascertainment biases cannot be excluded.

An additional limitation inherent in the pooling of case reports and case series is the limited availability and heterogeneity of long-term clinical follow-up. This is particularly relevant because RSV infection has been associated with long-term respiratory sequelae, including recurrent wheezing and asthma [4,20,201,202,203], while both RSV and hMPV may be followed by persistent impairment of cardiorespiratory function for several months after hospitalization, potentially resulting in reduced functional independence [2,13,127,131,155,204]. In patients experiencing such complex clinical courses, long-term, ideally multi-year, follow-up would be essential to determine whether the infection and the associated cardiac complications have persistent effects on cardiac function or are associated with increased long-term susceptibility to arrhythmias. The available case-based literature did not permit systematic assessment of these potential long-term cardiovascular sequelae.

Third, diagnostic approaches for RSV and hMPV infection have historically been highly heterogeneous. While most cases diagnosed nowadays benefit from RT-qPCR and RT-LAMP, in previous decades diagnosis relied on dIF/iIF, ELISA, immunohistochemical testing, and viral isolation in cell cultures. Each diagnostic method has its own diagnostic performance, which is, in turn, substantially influenced by the type of biological specimen tested (i.e., airway samples, sputum, nasopharyngeal specimens, and even myocardial tissue), particularly given the time-dependent nature of RSV and hMPV infections [205]. In other words, the limited number of cases ultimately included in our pooled analysis may reflect, at least in part, underdiagnosis of cardiac complications associated with these viral infections rather than their actual rarity. On the other hand, the limited availability and variable diagnostic performance of the methods used to investigate cardiac tissue may also have affected the sensitivity and specificity of detecting viral involvement in myocardial and/or pericardial specimens. Consequently, the very small number of cases in which myocardial and/or pericardial involvement by RSV or hMPV was directly documented should be interpreted with considerable caution. Moreover, the long timespan covered by the included reports encompasses major changes in diagnostic methods, cardiac imaging, clinical awareness, and respiratory-virus testing practices. These changes have been dramatically accelerated during and after the COVID-19 pandemic, and we can rule out that it eventually resulted in the increased detection and reporting of hMPV cases. Improvements in diagnostic availability may therefore have influenced case ascertainment and reporting and should be considered when interpreting chronological differences in the available evidence.

Fourth, the available studies did not provide consistent information on the RSV subtype associated with the reported cases. Despite their similarities, RSV-A and RSV-B exhibit substantial differences in their virological characteristics, with growing interest in the potential implications of such differences for the effectiveness of mAbs and currently available vaccines [4,20]. Moreover, although both RSV and hMPV have generally been regarded as highly or relatively stable pathogens [2,4,20,206,207]. Subtyping cases with documented cardiovascular involvement may help clarify whether specific viral strains exhibit a greater propensity for myocardial and/or pericardial involvement.

Fifth, the rapidly evolving clinical course of both cardiac disease and respiratory infection should be considered, as it may have hampered accurate characterization of some patient-specific features, potentially leading to misclassification or overestimation of certain clinical and individual characteristics. Given the limited size of our pooled sample, the incomplete characterization of even a small number of cases may have substantially affected the identification of specific clinical features and their distribution within the study population.

Sixth, the extreme heterogeneity in the age of the included cases, ranging from newborns to older adults aged >70 years, should also be considered. This is particularly relevant not only because of the markedly different distribution of lifestyle-related comorbidities, which are understandably uncommon among infants and children, but also, and perhaps more importantly, because of age-related differences in the ability to perceive, recognize, and report symptoms. Indeed, the clinical meaning of a symptom such as “dyspnea” is necessarily different when reported in a newborn compared with an adult. In infants and young children, symptom recognition largely depends on parents or other caregivers identifying changes in the child’s clinical status or behavior. Adults, in contrast, may directly perceive relatively subtle changes in their health status, including reduced exercise tolerance during mild or moderate physical activity, and seek medical attention accordingly. Therefore, differences in symptom reporting and recognition across age groups may have influenced the apparent clinical presentation of RSV- and hMPV-associated cardiac complications, as otherwise suggested by age-stratified analyses.

Seventh, the use of the CCI in a population encompassing both pediatric and adult patients represents an additional limitation. Although the index provided a pragmatic, standardized framework for summarizing heterogeneous comorbidity profiles, it was not specifically developed or validated for pediatric populations. Therefore, CCI-based comparisons, particularly those involving children and infants, should be interpreted as descriptive measures of reported comorbidity burden rather than validated estimates of prognosis or clinical complexity.

Eight, the total number of included cases was relatively small, comprising only 102 patients (71 RSV and 31 hMPV cases). Moreover, our analyses included a relatively large number of exploratory variables, and the very wide confidence intervals for several estimates indicate substantial statistical imprecision rather than precise estimates of clinical associations. Therefore, the findings from the binary logistic regression analyses should be interpreted with considerable caution. Similarly, the first two dimensions of the MCA accounted for only 33.1% of the total inertia. Consequently, the substantial overlap observed between RSV and hMPV cases in the MCA space should be regarded as an exploratory finding rather than as evidence of phenotypic equivalence between the two infections.

### 4.5. Future Research

Several research priorities emerge from the present findings. First, prospective studies based on systematically investigated cohorts of patients with laboratory-confirmed RSV and hMPV infection are needed to establish the actual incidence and spectrum of cardiac complications, which cannot be inferred from case reports. Standardized definitions of myocardial injury and cardiac complications, together with predefined diagnostic protocols and longitudinal follow-up, would improve comparability across studies. Second, adequately powered comparative studies should determine whether RSV and hMPV are associated with genuinely different cardiac phenotypes after accounting for age, comorbidities, disease severity, and coinfections. Third, the mechanisms underlying cardiac involvement remain insufficiently characterized. Future studies integrating virological, inflammatory, imaging, and, where available, histopathological data may help distinguish direct viral myocardial injury from systemic inflammatory, hypoxic, or hemodynamic mechanisms. Finally, longitudinal studies are required to characterize recovery of cardiac function and the potential persistence of arrhythmias, ventricular dysfunction, or other cardiovascular sequelae after the acute infection.

## 5. Conclusions

The present systematic review highlights that both RSV and hMPV infections can be associated with a broad spectrum of potentially severe cardiac complications, with myocarditis representing the predominant reported manifestation. The apparent differences between viral groups were strongly influenced by age, comorbidity, diagnostic work-up, coinfection, and selective publication. These data characterize the spectrum of reported presentations but cannot provide estimates of incidence, comparative risk, or pathogen-specific causality. Prospective, systematically investigated cohorts with standardized cardiac definitions of cardiac involvement and longitudinal follow-up are needed.

## Figures and Tables

**Table 1:** Summary of search strategy.

Source	RSV	Tot.	hMPV	Tot.
Pubmed First inquiry: 20 August 2024 Last inquiry: 14 August 2026	(“Respiratory Syncytial Viruses”[Mesh] OR “Respiratory Syncytial Virus Infections”[Mesh] OR “Respiratory Syncytial Virus, Human”[Mesh] OR RSV OR “respiratory syncytial virus”) AND (“Pericarditis”[Mesh] OR “Myocarditis”[Mesh] OR “Cardiac Tamponade”[Mesh] OR “Heart Failure”[Mesh] OR “Arrhythmias, Cardiac”[Mesh] OR “Myocardial Ischemia”[Mesh] OR “Coronary Artery Disease”[Mesh] OR “Heart Arrest”[Mesh] OR “Death, Sudden, Cardiac”[Mesh] OR “Heart Diseases”[Mesh] OR “Endocarditis”[Mesh])	492	(“Metapneumovirus”[Mesh] OR hMPV OR MPV OR “meta-pneumovirus” OR “metapneumovirus”) AND (“Pericarditis”[Mesh] OR “Myocarditis”[Mesh] OR “Cardiac Tamponade”[Mesh] OR “Heart Failure”[Mesh] OR “Arrhythmias, Cardiac”[Mesh] OR “Myocardial Ischemia”[Mesh] OR “Coronary Artery Disease”[Mesh] OR “Heart Arrest”[Mesh] OR “Death, Sudden, Cardiac”[Mesh] OR “Heart Diseases”[Mesh] OR “Endocarditis”[Mesh])	501
Scopus First inquiry: 20 August 2024 Last inquiry: 14 August 2026	(“Respiratory Syncytial Virus*” OR “Respiratory Syncytial Virus Infection*” OR “RSV”) AND (“Pericarditis” OR “Cardiac Tamponade” OR “Heart Failure” OR “Arrhythmias, Cardiac” OR “Myocardial Ischemia” OR “Coronary Artery Disease” OR “Heart Arrest” OR “Death, Sudden, Cardiac” OR “Heart Diseases” OR “Endocarditis”)	405	(“Metapneumovirus” OR hMPV OR MPV OR “meta-pneumovirus” OR “metapneumovirus”) AND (“Pericarditis” OR “Myocarditis” OR “Cardiac Tamponade” OR “Heart Failure” OR “Arrhythmias, Cardiac” OR “Myocardial Ischemia” OR “Coronary Artery Disease” OR “Heart Arrest” OR “Death, Sudden, Cardiac” OR “Heart Diseases” OR “Endocarditis”)	415
Embase First inquiry: 20 August 2024 Last inquiry: 14 August 2026	(‘human respiratory syncytial virus’/exp OR ‘human respiratory syncytial virus’ OR ‘respiratory syncytial virus’) AND (‘pericarditis’ OR ‘heart tamponade’ OR ‘heart failure’ OR ‘heart arrhythmia’ OR ‘heart muscle ischemia’ OR ‘coronary artery disease’ OR ‘heart arrest’ OR ‘myocarditis’ OR ‘virus myocarditis’ OR ‘endocarditis’)	1518	(‘human metapneumovirus’ OR ‘human metapneumovirus infection’) AND (‘pericarditis’ OR ‘heart tamponade’ OR ‘heart failure’ OR ‘heart arrhythmia’ OR ‘heart muscle ischemia’ OR ‘coronary artery disease’ OR ‘heart arrest’ OR ‘myocarditis’ OR ‘virus myocarditis’ OR ‘endocarditis’)	165

**Table 2:** Summary of general characteristics of the sampled case reports on human metapneumovirus (hMPV) and respiratory syncytial virus (RSV) with documented cardiac complications (note: 95% CI = 95% confidence interval; For ease of interpretation, *p*-values < 0.05 are highlighted in bold; IQR = interquartile range).

Variable	hMPV (n/31, %)	RSV (n/71, %)	*p*-Value
Gender			0.748
Male	17, 54.8%	43, 60.6%	
Female	14, 45.2%	28, 39.4%	
Age (years)			**<0.001**
Average (95% CI)	36.7 (25.6; 47.8)	16.8 (9.5; 24.0)	
Median (IQR)	41.5 (3.5; 63.0)	1.5 (0.2; 31.0)	
Age < 18 years	12, 38.7%	53, 74.6%	**<0.001**
≤30 days	0, -	17, 23.9%	**<0.001**
1–11 months	5, 16.1%	24, 33.8%	
12–59 months	4, 12.9%	11, 15.5%	
5–17 years	3, 9.7%	4, 5.6%	
18–44 years	3, 9.7%	4, 5.6%	
45–65 years	7, 22.6%	5, 7.0%	
More than 65 years	9, 29.0%	6, 8.5%	
Charlson Comorbidity Index (CCI)			**0.005**
No reported comorbidities	13, 41.9%	55, 77.5%	
Mild (CCI 1 to 2)	9, 29.0%	9, 12.7%	
Moderate (CCI 3 to 4)	5, 16.1%	3, 4.2%	
Severe (CCI ≥ 5)	4, 12.9%	4, 5.6%	
Features before hospital admission			
Days since onset of symptoms			0.400
Average (95% CI)	6.1 (3.6; 8.7)	4.9 (3.4; 6.5)	
Median (IQR)	4.0 (2.0; 7.0)	3.0 (2.0; 5.0)	
Signs of respiratory syndrome			0.709
LRTI	20, 64.5%	48, 67.6%	
URTI/ILI	9, 29.0%	16, 22.5%	
Unspecific features	2, 6.5%	7, 9.9%	
Fever (temperature ≥ 38.0 °C)	21, 67.7%	40, 56.3%	0.389
Shortness of breath	27, 87.1%	47, 66.2%	0.053
Dyspnea	29, 93.5%	47, 66.2%	**0.008**
Chest pain	13, 41.9%	13, 18.3%	**0.023**
Cough	24, 77.4%	40, 56.3%	0.071
Sneezing	8, 25.8%	21, 29.6%	0.696
Rhinorrhea	4, 12.9%	27, 38.0%	**0.021**
Asthenia	17, 54.8%	32, 45.1%	0.488
Arrhythmia			
Pre-hospital workout	2, 6.5%	12, 16.9%	0.272
At admission	9, 29.0%	40, 56.3%	**0.011**
Convulsions	2, 6.5%	3, 4.2%	1.000
Lethargy/Altered mental status	5, 16.1%	24, 33.8%	0.114
Cardiac arrest	4, 12.9%	13, 18.3%	0.700

**Table 3:** Clinical features and main outcomes of the sampled case reports on human metapneumovirus (hMPV) and respiratory syncytial virus (RSV) with documented cardiac complications (note: ECMO = extracorporeal membrane oxygenation; CPAP = continuous positive airway pressure; ICU = intensive care unit; 95% CI = 95% confidence interval; For ease of interpretation, *p*-values < 0.05 are highlighted in bold).

Variable	hMPV (n/31, %)	RSV (n/71, %)	*p*-Value
Heart Rate			0.131
Normal	7, 22.6%	20, 28.2%	
Tachycardia	23, 74.2%	38, 53.5%	
Bradycardia	1, 3.2%	7, 9.9%	
Cardiac arrest	0, -	6, 8.5%	
Blood Pressure			0.094
Normal	19, 61.3%	44, 62.0%	
Hypertension	3, 9.7%	1, 1.4%	
Hypotension	9, 29.0%	20, 28.2%	
Cardiac arrest	0, -	6, 8.5%	
Respiratory rate			0.095
Normal	15, 48.4%	41, 57.7%	
Increased	16, 51.6%	24, 33.8%	
Cardiac arrest	0, -	6, 8.5%	
Temperature			0.087
Normal range (36.0 °C to 38.0 °C)	19, 61.3%	51, 71.8%	
Fever (>38.0 °C)	11, 35.5%	15, 21.1%	
Hypothermia (<36.0 °C)	1, 3.2%	0, -	
Not assessed due to cardiac arrest	0, -	5, 7.0%	
Signs of Encephalopathy	3, 9.7%	5, 7.0%	0.956
Signs of Deep Vein Thrombosis	3, 9.7%	1, 1.4%	0.154
Signs of Diffuse Alveolar Hemorrhage	4, 12.9%	2, 3.0%	0.147
Cardiac diagnoses			
Myocarditis	27, 87.1%	53, 74.6%	0.253
Pericarditis	8, 25.8%	12, 16.9%	0.441
Endocarditis	1, 3.2%	1, 1.4%	1.000
Heart block	6, 19.4%	16, 22.5%	0.922
Heart failure	15, 48.4%	28, 39.4%	0.533
Infarction	1, 3.2%	1, 1.4%	1.000
Coinfections			
Any pathogen	8, 25.0%	1, 1.4%	**<0.001**
Any bacteria	6, 19.4%	1, 1.4%	**0.004**
*S. pneumoniae*	3, 9.7%	0, -	**0.043**
Any viruses other than hMPV or RSV	3, 9.7%	0, -	**0.043**
Need for respiratory support			
O_2_ high flow	24, 74.2%	47, 67.1%	0.635
CPAP	16, 51.6%	35, 50.0%	1.000
Mechanical Ventilation	19, 61.3%	31, 44.3%	0.174
ECMO	6, 19.4%	6, 8.6%	0.226
Outcomes			
Hospital stay (days)			0.988
Average (95% CI)	18.2 (10.0; 26.4)	14.8 (11.7; 17.9)	
Median (IQR)	14.0 (7.0; 20.0)	13.0 (6.5; 18.5)	
ICU admission	30, 96.8%	55, 77.5%	0.034
Recovery	21, 67.7%	49, 69.0%	0.299
Residual disease	1, 3.2%	8, 11.3%	
Death	9, 29.0%	14, 19.7%	

**Table 4:** Main electrographic features of the sampled case reports on human metapneumovirus (hMPV) and respiratory syncytial virus (RSV) with documented cardiac complications.

Main Features	hMPV (n/31, %)	RSV (n/71, %)	*p*-Value
Rhythm anomalies			0.374
Non-rhythm anomalies (sinus rhythm)	11, 35.5%	19, 26.8%	
Any rhythm anomaly	20, 64.5%	52, 73.2%	
Detail of rhythm anomalies			0.181
Tachycardia (any)	7, 22.6%	20, 28.2%	
Atrial fibrillation/flutter	3, 9.7%	3, 4.2%	
Atrial tachycardia	0, -	4, 5.6%	
Sinus tachycardia	3, 9.7%	6, 8.5%	
Supraventricular tachycardia	1, 3.2%	6, 8.5%	
Ventricular tachycardia	0, -	1, 1.4%	
Heart blocks (any)	0, -	11, 15.5%	
Atrio-ventricular	0, -	9, 12.7%	
Sinoatrial block	0, -	2, 2.8%	
Bradycardia (any)	4, 16.5%	9, 12.7%	
Sinus bradycardia	2, 6.5%	2, 2.8%	
Asystole	2, 10.0%	4, 7.5%	
Mixed rhythms	0, -	3, 4.2%	
Detail of ECG track anomalies			
ST segment	10, 32.3%	24, 34.3%	1.000
Inverted T wave	2, 6.5%	10, 14.3%	0.430
QRS anomalies (any)	4, 12.9%	10, 14.3%	1.000

**Table 5:** Main chest X-ray features of the sampled case reports on human metapneumovirus (hMPV) and respiratory syncytial virus (RSV) with documented cardiac complications.

Main Features	hMPV	RSV	*p*-Value
CXR	n/23, %	n/39, %	
Enlarged heart volumes	7, 30.4%	19, 48.7%	0.253
Signs of edema	7, 30.4%	15, 38.5%	0.716
Any opacities	13, 56.5%	19, 48.7%	0.741
Infiltrative lesions	11, 47.8%	24, 61.5%	0.431
CT scans	n/17, %	n/8, %	
Any heart lesion	4, 23.5%	2, 25.0%	1.000
Infiltrative lesions	11, 73.3%	5, 62.5%	0.951
Pleural effusions	8, 50.0%	6, 75.0%	0.464
MRI	n/7, %	n/1, %	
Any heart lesion	6, 85.7%	1, 100%	1.000
Ultrasonographic features	n/24, %	n/48, %	
Any sign of pericarditis	10, 41.7%	19, 39.6%	1.000
Any sign of myocarditis	20, 83.3%	41, 85.4%	1.000
Any sign of endocarditis	1, 4.2%	1, 2.1%	1.000
Diagnosis of Takotsubo	3, 12.5%	0, -	0.061
Ejection Fraction		1	0.450
Normal (>50%)	6, 25.0%	14, 29.2%	
Depressed (50% to 30%)	8, 33.3%	21, 43.8%	
Severely depressed (<30%)	10, 41.7%	13, 27.1%	

**Table 6:** Histopathologic findings of myocardial tissue from sampled patients (Note: RSV = respiratory syncytial virus).

Reference	Pathogen	Sampling	Pathogen Identified Within the Sample	Histopathology
Araki et al. (Kawashima et al., pt. 2) [79]	RSV	Biopsy	No	Not available
Bairan et al. 1974 [62]	RSV	Biopsy	No	Thickened epicardium
Eng et al. 2023 [66]	RSV	Biopsy	No	Lymphocytic myocarditis
Gavotto et al. 2019 [70]	RSV	Autopsy	Yes	Lymphocytic myocarditis
Goto et al. (Kawashima et al. 2021, pt. 12) [79]	RSV	Autopsy	No	Myocarditis
Karatza et al. 2017 [28]	RSV	Biopsy	No	Mild fibrosis Scarce lymphocytic infiltrate
Kawashima et al. 2021, pt. 7 [79]	RSV	Biopsy	No	Myocarditis
Rubach et al. 2013 [41]	RSV	Biopsy	Yes	Fibrinous pericarditis
Salfi et al. 2024 [95]	RSV	Biopsy	No	Mild and focal lymphocytic myocarditis without necrosis
Takamizawa et al. 2022 [121]	hMPV	Biopsy	No	Mild myocardial fibrosis

**Table 7:** Sensitivity analysis of clinical characteristics and outcomes of human metapneumovirus (hMPV) and respiratory syncytial virus (RSV) cases according to age group. Data are presented separately for patients aged <1 year, 1–17 years, and ≥18 years. Comparisons were performed between hMPV and RSV cases within each age stratum by means of chi-squared test for categorical data and with Mann–Whitney U test for continuous variables. Data on ultrasonographic features are reported for available cases. Note: LRTI = lower respiratory tract infection; URTI = upper respiratory tract infection; CCI = Charlson comorbidity index; EF = ejection Fraction; CPAP = continuous positive airway pressure; ECMO = extracorporeal membrane oxygenation; ICU = intensive care unit. For ease of interpretation, *p*-values < 0.05 are highlighted in bold.

	Age <1 Year			Age 1 to 17 Years			18 Years and Older		
	hMPV (n/5, %)	RSV (n/41, %)	*p*-Value	hMPV (n/7, %)	RSV (n/15, %)	*p*-Value	hMPV (n/19, %)	RSV (n/15, %)	*p*-Value
Gender			0.598			1.000			1.000
Male	2, 40.0%	26, 63.4%		3, 42.9%	8, 53.3%		12, 63.2%	9, 60.0%	
Female	3, 60.0%	15, 35.6%		4, 57.1%	7, 46.7%		7, 36.8%	6, 40.0%	
General features			0.808			0.533			0.375
LRTI	4, 80.0%	29, 70.7%		4, 57.1%	12, 80.0%		12, 63.2%	7, 46.7%	
URTI	1, 20.0%	9, 22.0%		2, 28.6%	2, 13.3%		6, 31.6%	5, 33.3%	
Negative	-	3, 7.3%		1, 14.3%	1, 6.7%		1, 5.3%	3, 20.0%	
Symptoms									
Chest pain	0, -	1, 2.4%	1.000	2, 28.6%	2, 13.3%	0.787	11, 57.9%	10, 66.7%	0.867
Dyspnea	3, 60.0%	27, 65.9%	1.000	7, 100%	8, 53.3%	0.090	19, 100%	12, 80.0%	0.152
Shortness of breath	5, 100%	25, 61.0%	0.218	7, 100%	11, 73.3%	0.359	15, 78.9%	11, 73.3%	1.000
Asthenia/severe fatigue	1, 20.0%	13, 31.7%	0.982	3, 42.9%	8, 53.3%	1.000	13, 68.4%	11, 73.3%	1.000
Any arrhythmia	0, -	8, 19.5%	0.644	0, -	2, 13.3%	0.828	2, 10.5%	2, 13.3%	1.000
Cough	4, 80.0%	18, 43.9%	0.293	4, 57.1%	9, 60.0%	1.000	16, 84.2%	13, 86.7%	1.000
Sneezing	2, 40.0%	7, 17.1%	0.533	0, -	5, 33.3%	0.233	6, 31.6%	9, 60.0%	0.190
Rhinorrhea	1, 20.0%	14, 34.1%	0.895	1, 14.3%	6, 40.0%	0.475	2, 10.5%	7, 46.7%	**0.048**
Fever (>38 °C)	5, 100%	18, 43.9%	0.058	3, 42.9%	11, 73.3%	0.364	13, 68.4%	11, 73.3%	1.000
Altered mental status/lethargy	1, 20.0%	14, 34.1%	0.895	0, -	7, 46.7%	0.090	4, 21.1%	3, 20.0%	1.000
Convulsion	1, 20.0%	0, -	0.204	1, 14.3%	2, 13.3%	1.000	0, -	1, 6.7%	0.904
Cardiac arrest	3, 60.0%	5, 12.2%	**0.042**	1, 14.3%	6, 40.0%	0.475	0, -	2, 13.3%	0.365
CCI			1.000			0.321			0.933
Negative	5, 100%	38, 92.7%		4, 57.1%	13, 86.7%		4, 21.1%	4, 26.7%	
CCI 1–2	0, -	3, 7.3%		3, 42.9%	2, 13.3%		6, 31.6%	4, 26.7%	
CCI 3–4	0, -	0, -		0, -	0, -		5, 26.3%	3, 20.0%	
CCI 5+	0, -	0, -		0, -	0, -		4, 21.1%	4, 26.7%	
Any coinfection	3, 60.0%	0, -	**<0.001**	3, 42.9%	1, 6.7%	0.145	2, 10.5%	0, -	0.575
Heart rate			0.883			0.297			0.055
Normal	1, 20.0%	10, 24.4%		2, 28.6%	2, 13.3%		4, 21.1%	8, 53.5%	
Increased	4, 80.0%	27, 65.9%		4, 57.1%	5, 33.3%		15, 78.9%	6, 40.0%	
Decreased	0, -	3, 7.3%		1, 14.3%	3, 20.0%		0, -	1, 6.7%	
Cardiac arrest before assessment	0, -	1, 2.4%		0, -	5, 33.3%		0, -	0, -	
Respiratory rate			0.583			0.168			0.060
Normal	4, 80.0%	23, 56.1%		3, 42.9%	6, 40.0%		8, 42.1%	12, 80.0%	
Increased	1, 20.0%	17, 41.5%		4, 57.1%	4, 26.7%		11, 57.9%	3, 20.0%	
Cardiac arrest before assessment	0, -	1, 2.4%		0, -	5, 33.3%		0, -	0, -	
Arterial pressure			0.694			0.165			0.646
Normal	4, 80.0%	25, 61.0%		4, 57.1%	8, 53.3%		11, 57.9%	11, 73.3%	
Increased	0, -	0, -		1, 14.3%	0, -		2, 10.5%	1, 6.7%	
Decreased	1, 20.0%	15, 36.6%		2, 28.6%	2, 13.3%		6, 31.6%	3, 20.0%	
Cardiac arrest before assessment	0, -	1, 2.4%		0, -	5, 33.3%		0, -	0, -	
Temperature			0.841			0.226			0.867
Normal	4, 80.0%	35, 85.4%		4, 57.1%	6, 40.0%		11, 57.9%	10, 66.7%	
Increased	1, 20.0%	5, 12.2%		2, 28.6%	5, 33.3%		8, 42.1%	5, 33.3%	
Decreaed	0, -	0, -		0, -	4, 26.7%		0, -	0, -	
Cardiac arrest before assessment	0, -	1, 2.4%		1, 14.3%	0, -		0, -	0, -	
Ultrasonographic assessment of EF			0.131			0.108			0.195
Normal	0, -	13, 43.3%		2, 40.0%	0, -		4, 23.5%	1, 9.1%	
30 to 50%	2, 100%	9, 30.0%		1, 20.0%	5, 71.4%		5, 29.4%	7, 63.6%	
<30%	0, -	8, 26.7%		2, 40.0%	2, 28.6%		8, 47.1%	3, 27.3%	
Signs of									
Myocarditis	5, 100%	28, 68.3%	0.337	6, 85.7%	12, 80.0%	1.000	16, 84.2%	13, 86.7%	1.000
Pericarditis	0, -	4, 9.8%	1.000	2, 28.6%	4, 26.7%	1.000	6, 31.6%	4, 26.7%	1.000
Endocarditis	0, -	1, 2.4%	1.000	0, -	0, -	-	1, 5.3%	0, -	1.000
Heart block	3, 60.0%	7, 17.1%	0.105	2, 28.6%	6, 40.0%	0.966	1, 5.3%	3, 20.0%	0.431
Heart failure	4, 80.0%	16, 39.0%	0.205	6, 86.7%	5, 33.3%	0.067	5, 26.3%	7, 46.7%	0.383
Infarction	0, -	0, -	-	0, -	0, -	-	1, 5.3%	1, 6.7%	1.000
Respiratory support									
O2 therapy	4, 80.0%	32, 78.0%	1.000	5, 71.4%	8, 57.1%	0.874	14, 73.7%	7, 46.7%	0.210
CPAP	4, 80.0%	24, 58.5%	0.658	5, 71.4%	5, 35.7%	0.280	7, 36.8%	6, 40.0%	1.000
Ventilatory support	4, 80.0%	25, 61.0%	0.733	6, 85.7%	5, 35.7%	0.089	9, 47.4%	5, 33.3%	0.635
ECMO	0, -	2, 4.9%	1.000	4, 57.1%	1, 7.1%	0.046	2, 10.5%	3, 20.0%	0.774
ICU admission	5, 100%	35, 85.4%	0.831	7, 100%	9, 60.0%	0.148	18, 94.7%	11, 73.3%	0.207
Hospital stay (days; average, 95% CI)	13.0 (0.3; 25.7)	14.4 (10.7; 18.0)	0.808	14.3 (3.0; 25.6)	12.2 (5.3; 19.1)	0.591	20.9 (7.9; 34.0)	17.8 (9.1; 26.4)	0.708
Death	3, 60.0%	8, 19.5%	0.147	3, 42.9%	5, 33.3%	1.000	3, 15.8%	1, 6.7%	0.777

## Data Availability

Raw data are available from Supplementary Materials.

## Supplementary Materials

The following supporting information can be downloaded at: https://www.mdpi.com/article/10.3390/v18101106/s1, File S1; PRISMA checklist of the present study; File S2: raw data included in the analyses.

## Abbreviations

**ACIP**	Advisory Committee on Immunization Practices
**aOR**	Adjusted odds ratio
**CCI**	Charlson Comorbidity Index
**CHD**	Congenital heart disease
**CI**	Confidence interval
**CPAP**	Continuous positive airway pressure
**CT**	Computed tomography
**CXR**	Chest X-ray
**dIF**	Direct immunofluorescence
**ECG**	Electrocardiography/electrocardiogram
**ECMO**	Extracorporeal membrane oxygenation
**EF**	Ejection fraction
**ELISA**	Enzyme-linked immunosorbent assay
**hMPV**	Human metapneumovirus
**ICU**	Intensive care unit
**iIF**	Indirect immunofluorescence
**IHD**	Ischemic heart disease
**ILI**	Influenza-like illness
**LRTI**	Lower respiratory tract infection
**LV**	Left ventricular/left ventricle
**mAb/mAbs**	Monoclonal antibody/monoclonal antibodies
**MCA**	Multiple correspondence analysis
**MRI**	Magnetic resonance imaging
**OR**	Odds ratio
**PRISMA**	Preferred Reporting Items for Systematic Reviews and Meta-Analyses
**RCT**	Randomized controlled trial
**RNA**	Ribonucleic acid
**RSV**	Respiratory syncytial virus
**RT-LAMP**	Reverse transcription loop-mediated isothermal amplification
**RT-PCR**	Reverse transcription polymerase chain reaction
**RT-qPCR**	Reverse transcription quantitative polymerase chain reaction
**STIKO**	Standing Committee on Vaccination (*Ständige Impfkommission*)
**URTI**	Upper respiratory tract infection
**VE**	Vaccine effectiveness

## Appendix A

**Table A1:** Working definition of conditions included in the main analysis.

Item		Working Definition
Heart disease	Investigated result	Any type of disease affecting the heart, including coronary heart disease, arrhythmias, heart failure, and valve disease.
Cardiac Arrest	Investigated result	Sudden loss of all heart activity due to an irregular heart rhythm in a person who may or may not have been previously diagnosed with heart disease.
Cardiogenic shock	Investigated result	Severe impairment of myocardial performance that results in diminished cardiac output, end-organ hypoperfusion and hypoxia [208]. For the aims of the present review, it also includes heart block and ventricular fibrillation.
Tamponade	Investigated result	Condition resulting from the accumulation of pericardial fluid under pressure, leading to impaired cardiac filling and hemodynamic compromise [209]
Myocarditis	Investigated result	Inflammatory cardiomyopathy associated with any cardiac dysfunction [210]
Heart failure	Investigated result	A syndrome caused by any impairment in the heart’s ability to fill with and pump blood due to any disorder of the myocardial tissue [211]
Pericarditis	Investigated result	Any inflammation of the pericardium, including both the acute form (i.e., a new-onset inflammation lasting < 4 to 6 weeks) and the chronic one, characterized by chest pain, pericardial friction rub, electrocardiographic changes, with/without pericardial effusion [212]
Pancarditis	Investigated result	A condition characterized by the combined evidence of endocarditis, myocarditis and pericarditis [213].
Rhythm anomaly	Investigated result	Any problem in the rate and/or rhythm of a person’s heartbeat, not including (for the aims of the present review) heart block and ventricular fibrillation.
Ischemic disorder	Investigated result	Any condition characterized by reduced cardiac blood flow that causes a mismatch between myocardial oxygen supply and demand [214].
Respiratory support	Investigated result	Any process where a ventilator replaces or support the patient’s own breathing, including invasive mechanical ventilation, non-invasive ventilation, and limitedly to the aims of the present study, continuous positive airway pressure, but excluding nasal high-flow therapy.
Mechanical circulatory support	Investigated result	Any mechanical device which can be added to the physiological circulation in order to support or replace the heart and/or the lungs [215].
Recovery	Outcome	No evidence of residual disorder(s) and/or long-term medical complications due to the RSV- or hMPV- induced clinical syndrome.
Residual disease	Outcome	Any evidence of residual disorder(s) and/or long-term medical complications due to the RSV- or hMPV- induced clinical syndrome.

**Table A2:** Characteristics of 102 subjects with diagnosis of any sign of heart disease associated with respiratory syncytial virus (RSV) and/or human metapneumovirus (hMPV) infections, and methodological quality of included cases rates in accordance with Murad et al. [54]. Items D5 and D6 were removed as they were mostly relevant for the analysis of adverse drug events. Note: AW = airways; NA = not available; LV = left ventricle; LRTI = lower respiratory tract infection; URTI = upper respiratory tract infection; RD = rhythm disorder; Peric. = pericarditis; Myo. = myocarditis; CHD = congestive heart disease; IHD = ischemic heart disease; ALPACA = Anomalous origin of the left coronary artery from the pulmonary artery; RT-PCR = real-time polymerase chain reaction; RT-LAMP = real-time loop-mediated isothermal amplification; ELISA = enzyme-linked immunosorbent assay; IF = immunofluorescence.

Study	Year	Sex, Age	Country	Pathogen	Clinical Features		Echo	Diagnosis		Outcome	Quality Assessment (Sum Score-/6)
					Respiratory	Cardiac		Assay	Myocardium		
Abdulghani et al. [58]	2025	M, 3 d	Saudi Arabia	RSV	LRTI	RD	NA	AW, RT-PCR	NA	Recovery	6
Al Hennawi et al. (pt. 1) [100]	2024	M, 68 y	USA	hMPV	LRTI	RD	Reduced LV function	AW, RT-PCR	NA	Recovery	6
Al Hennawi et al. (pt. 2) [100]	2024	F, 58 y	USA	hMPV	LRTI	RD	Reduced LV function	AW, RT-PCR	NA	Recovery	6
Almajed et al. [59]	2022	M, 42 y	USA	RSV	URTI	Peric.	Normal	AW, RT-PCR	NA	Recovery	6
Anstett et al. [101]	2013	M, 67 y	USA	hMPV	LRTI	Myo.	NA	AW, RT-PCR	NA	Recovery	6
Araki et al. (Kawashima et al., pt. 2) [79]	2012	M, 1 m	Japan	RSV	URTI	RD	Normal	AW, RT-LAMP	Neg.	Recovery	3
Armstrong and Menahem [60]	1993	M, 10 d	Australia	RSV	LRTI	RD	Normal	AW, IF	NA	Recovery	6
Aydogan et al. [61]	2022	M, 17 d	Turkey	RSV	LRTI	RD	Normal	AW, RT-PCR	NA	Recovery	6
Bairan et al. [62]	1974	M, 3 y	USA	RSV	LRTI	RD	NA	AW, Culture	Neg.	Recovery	4
Bhatia et al. [102]	2023	M, 53	India	hMPV	LRTI	Myo.	Reduced LV function	AW, RT-PCR	NA	Recovery	6
Campbell et al.[63]	2025	M, 60 y	USA	RSV	LRTI	Myo.	Reduced LV function	AW, RT-PCR	NA	Recovery	6
Castillo-Morales et al. [103]	2025	F, 40 y	Colombia	hMPV	LRTI	Myo.	Reduced LV function	AW, RT-PCR	NA	Death	6
Choi et al. [104]	2016	F, 76 y	Republic of Korea	hMPV	ILI	Myo.	Reduced LV function	AW, RT-PCR	NA	Recovery	6
Dabbah et al. [64]	2013	M, 1 m	Israel	RSV	LRTI	Myo.	Normal	AW, RT-PCR	NA	Recovery	6
David et al. [105]	2009	M, 62 y	USA	hMPV	LRTI	Peric.	Normal	AW, RT-PCR	NA	Recovery	6
Donnerstein et al. (pt. 1) [65]	1994	M, 1 m	USA	RSV	LRTI	RD	Normal	AW, Culture	NA	Recovery	6
Donnerstein et al. (pt. 2) [65]	1994	F, 5 m	USA	RSV	LRTI	RD	NA	AW, IF Culture	NA	Death	6
Donnerstein et al. (pt. 4) [65]	1994	M, 1 m	USA	RSV	LRTI	RD	Normal	AW, IF Culture	NA	Recovery	6
Donnerstein et al. (pt. 5) [65]	1994	M, 16 d	USA	RSV	LRTI	RD	Normal	AW, IF Culture	NA	Recovery	6
Donnerstein et al. (pt. 7) [65]	1994	M, 11 m	USA	RSV	LRTI	RD	NA	AW, IF Culture	NA	Residual Disease	6
Donnerstein et al. (pt. 8) [65]	1994	M, 12 d	USA	RSV	LRTI	RD	Normal	AW, IF Culture	NA	Recovery	6
Eng et al. [66]	2023	F, 63 y	USA	RSV	LRTI	Myo.	Depressed biventricular function	NA	Neg.	Recovery	6
Erdogan et al. [37]	2019	F, 7 y	Turkey	RSV	LRTI	Myo.	Mitral and Tricuspid regurgitation	AW, RT-PCR	NA	Recovery	6
Fox et al. [67]	2012	F, 37 y	Germany	RSV	URTI	Myo.	Reduced LV function	AW, antigen	NA	Recovery	6
Garcia Suarez et al. [68]	2000	M, 18 m	Spain	RSV	URTI	Myo.	LV dilatation, reduced contractility	AW, ELISA, culture	NA	Recovery	6
Garrido Ocaña et al. (pt. 1) [69]	2011	F, 11 d	Spain	RSV	LRTI	RD	Normal	AW, Antigen	NA	Recovery	6
Garrido Ocaña et al. (pt. 2) [69]	2011	F, 18 d	Spain	RSV	LRTI	RD	Normal	AW, Antigen	NA	Recovery	6
Gavotto et al. [70]	2019	F, 3 y 11 m	France	RSV	Neg.	Myo.	NA	Tissue PCR	Pos.	Death	6
Giles and Gohd (pt. 1) [71]	1976	M, 15 y	USA	RSV	LRTI	RD	Bilateral enlargement of ventricles,	Serum, IF	NA	Recovery	6
Giles and Gohd (pt. 2) [71]	1976	M, 23 y	USA	RSV	URTI	CHD	NA	Serum, IF	NA	Recovery	6
Gopalan et al. [107]	2024	M, 27 y	USA	hMPV	URTI	Myo./Peric.	Reduced LV function	A, RT-PCR	NA	Recovery	3
Goto et al. (Kawashima et al., pt. 12) [79]	2007	M, 1 y	Japan	RSV	URTI	RD	NA	AW, RT-LAMP	Pos.	Recovery	6
Haddad et al. [72]	2012	M, 3 m	USA	RSV	LRTI	Myo.	Normal	AW, IF	NA	Death	6
Huang et al. [73]	1998	M, 7 w	USA	RSV	URTI	Myo.	Reduced LV function, mitral regurgitation	AW, Antigen	NA	Recovery	6
Hutchison et al. [74]	1994	M, 10 w	USA	RSV	LRTI	Peric.	Poor myocardial contractility	AW, Antigen	NA	Recovery	6
Imamura et al. [75]	2021	F, 1 y	Japan	RSV	LRTI	CHD	Diffuse hypokinesis	AW, RT-PCR	NA	Recovery	6
Ingram et al. [108]	2024	M, 43 y	USA	hMPV	LRTI	RD/Myo.	Reduced LV function	AW, RT-PCR	NA	Recovery	3
Ishikawa et al. (Kawashima pt. 10) [79]	2021	M, 1 m	Japan	RSV	URTI	Myo.	Myocarditis and heart failure	AW, RT-PCR	NA	Recovery	6
Jaber et al. [76]	2025	M, 26 d	Palestine	RSV	LRTI	Myo.	Mild left atrial and ventricular dilation with reduction in ejection fraction and mild to moderate mitral regurgitation	AW, RT-PCR	NA	Death	6
Jasti et al. [77]	2023	M, 31 y	USA	RSV	Neg.	IHD	Diffuse coronary stenosis	AW, RT-PCR	NA	Recovery	6
Jingu et al. [109]	2026	F, 40 y	Japan	hMPV	LRTI	Myo.	Takotsubo Syndrome	AW, RT-PCR	NA	Recovery	6
Jiun et al. [110]	2025	F, 67 y	Malaysia	hMPV	LRTI	Myo.	Signs of pericarditis	AW, RT-PCR	NA	Recovery	6
Kaburaki et al. [111]	2026	M, 64 y	Japan	hMPV	LRTI	Myo.	Severely depressed heart function	AW, RT-PCR	NA	Recovery	6
Kakimoto et al. (pt. 1) [78]	2016	M, 19 m	Japan	RSV	LRTI	Cardiac arrest	NA	AW, RT-PCR	NA	Recovery	6
Kakimoto et al. (pt. 2) [78]	2016	F, 19 m	Japan	RSV	LRTI	Cardiac arrest	NA	AW, RT-PCR	NA	Death	6
Karatza et al. [28]	2017	M, 10 m	Greece	RSV	URTI	Myo.	Mild tricuspid and pulmonary valve regurgitation	AW, Antigen	Neg.	Residual Disease	3
Kawashima et al. (pt. 11) [79]	2021	F, 12 m	Japan	RSV	URTI	Myo.	Depressed heart function, signs of pericarditis, effusion, cardiac enlargement	AW, Rapid test	NA	Recovery	3
Kawashima et al. (pt. 14) [79]	2021	F, 3 y	Japan	RSV	LRTI	CHD	Reduced myocardial function, mitral regurgitation	AW, RT-LAMP	NA	Residual Disease	3
Kawashima et al. (pt. 5) [79]	2021	F, 5 m	Japan	RSV	LRTI	CHD	Reduced myocardial function, mitral regurgitation	AW, RT-LAMP	NA	Residual Disease	3
Kawashima et al. (pt. 7) [79]	2021	F, 8 m	Japan	RSV	URTI	Myo.	Reduced LV function	AW, RT-LAMP	Neg	Recovery	3
Kawashima et al. (pt. 8) [79]	2021	F, 9 m	Japan	RSV	LRTI	CHD	Reduced left ventricular function, left ventricular enlargement	AW, RT-LAMP	NA	Death	6
Kayiran et al. [80]	2014	F, 5 m	Turkey	RSV	URTI	IHD	Reduced LV function, mild ventricular regurgitation, ALPACA	AW, Antigen	NA	Recovery	6
Kongala et al. [112]	2025	F, 59 y	USA	hMPV	Neg.	Cardiac Arrest Myo.	Takotsubo, severely depressed heart function	AW, RT-PCR	NA	Recovery	6
Krivonos et al. [113]	2025	M, 3 y	Russia	hMPV	LRTI	Myo.	NA	AW, RT-PCR	NA	Recovery	6
Lademann et al. [114]	2020	F, 4 y	Germany	hMPV	URTI	Myo.	Signs of heart failure	AW, RT-PCR	NA	Death	6
Makhlouf et al. [115]	2022	F, 14 y	France (La Réunion)	hMPV	URTI	Myo./ Peric.	Severely depressed heart function	AW, RT-PCR	NA	Death	6
Mehta et al. [116]	2020	M, 46 y	USA	hMPV	URTI	Myo.	Depressed heart function	AW, RT-PCR	NA	Recovery	3
Menahem and Uren [82]	1985	M, 3 y	Australia	RSV	LRTI	CHD	NA	AW, IF	NA	Residual Disease	6
Menahem S [81]	1991	F, 7 w	Australia	RSV	URTI	Myo.	Reduced fractional shortening	AW, IF	NA	Recovery	6
Menchise A [83]	2011	F, 7 m	USA	RSV	LRTI	RD	Reduced LV function	AW, RT-PCR	NA	Recovery	6
Mihyawi et al. [117]	2020	M, 26 y	USA	RSV	URTI	Peric.	Signs of pericarditis/effusion	AW, RT-PCR	NA	Recovery	6
Milas et al. [38]	2017	M, 40 y	USA	RSV	URTI	RD	NA	AW, RT-PCR	NA	Recovery	6
Misirlioglu et al. [84]	2006	M, 12 d	Turkey	RSV	LRTI	Myo.	NA	Tissue, Culture	NA	Recovery	6
Miura et al. [40]	2018	F, 4 y	Japan	RSV	LRTI	RD	Reduced LV function, mitral and tricuspid regurgitation	AW, Serum, RT-PCR	NA	Recovery	3
Mizuno et al. (Kawashima et al., pt. 13) [79]	2016	F, 2 y	Japan	RSV	URTI	RD	Ventricular wall thickening	AW, RT-LAMP	NA	Recovery	6
Monteiro de Almeida Magalhaes et al. [85]	2023	M, 16 y	Brazil	RSV	LRTI	Myo.	Reduced LV function	AW, RT-PCR	NA	Recovery	6
Mustafa et al. [86]	2025	F, 85 y	USA	RSV	LRTI	Myo.	Severely depressed ventricular function with no regional wall motion abnormalities	AW, not specified	NA	Recovery	6
O’Brien et al. [39]	2021	M, 83 y	USA	RSV	LRTI	RD	NA	AW, RT-PCR	NA	Residual Disease	6
Olesch and Bullock [87]	1998	F, 14 d	Australia	RSV	LRTI	Myo.	Normal	AW, IF	NA	Recovery	4
Oulego-Erroz et al. [88]	2021	F, 1 m	Spain	RSV	LRTI	Myo.	Reduced LV function	AW, serum, RT-PCR	NA	Residual Disease	3
Owensby et al. [118]	2024	F, 11 y	USA	hMPV	LRTI	Myo.	Depressed ventricular function	AW, not specified	NA	Recovery	6
Patel et al. [89]	2019	F, 31 y	USA	RSV	Neg.	Myo.	Reduced LV function	AW, RT-PCR	NA	Recovery	6
Playfor and Khader [90]	2005	M, 7 w	UK	RSV	LRTI	RD	Shunt (ostium secundum)	AW, ELISA	NA	Recovery	3
Puchkov et al. [91]	1972	M, 12 y	SSSR	RSV	LRTI	RD	NA	NA	NA	Recovery	4
Rubach et al. [41]	2013	M, 63 y	USA	RSV	LRTI	Peric.	Pericardial effusion, collapse of right ventricle	Tissue, histopathology	Neg.	Death	4
Ramzali et al. (pt. 1) [92]	2025	M, 7 m	Iran	RSV	LRTI	Myo.	NA	AW, rapid test	NA	Death	4
Ramzali et al. (pt. 3) [92]	2025	M, 7 m	Iran	hMPV	URTI	Myo	NA	AW, rapid test	NA	Death	4
Ramzali et al. (pt. 4) [92]	2025	F, 6 m	Iran	hMPV	LRTI	Myo	NA	AW, not specified	NA	Death	4
Ramzali et al. (pt. 6) [92]	2025	F, 11 m	Iran	hMPV	LRTI	Myo	NA	AW, rapid test	NA	Death	6
Ramzali et al. (pt. 7) [92]	2025	M, 5 y	Iran	hMPV	Neg.	Myo	NA	AW, rapid test	NA	Death	6
Raphael et al. [93]	2023	F, 9 m	Israel	RSV	URTI	RD	Neg.	AW, RT-PCR	NA	Recovery	6
Raphael et al. [93]	2023	M, 2 m	Israel	RSV	LRTI	RD	Neg.	AW, RT-PCR	NA	Recovery	6
Rethnaswamy et al. [125]	2025	M, 77 y	USA	hMPV	URTI	RD	Signs suspected for pericarditis	AW, RT-PCR	NA	Recovery	5
Rodriguez-Nava et al. [119]	2020	F, 62, y	USA	hMPV	LRTI	RD	NA	AW, RT-PCR	NA	Death	6
Royzman et al. [94]	2025	F, 73 y	USA	RSV	URTI	RD	NA	AW, RT-PCR	NA	Recovery	3
Rubach et al. [41,95]	2013	M, 63 y	USA	RSV	LRTI	Myo.	NA	Bioptic specimen	NA	Recovery	3
Sakha et al. (pt. 1) [36]	2004	M, 4 m	Iran	RSV	LRTI	Myo.	NA	AW, IF	NA	Death	3
Sakha et al. (pt. 2) [36]	2004	M, 10 m	Iran	RSV	LRTI	Myo.	Reduced LV function, reduced contractility	AW, IF	NA	Death	6
Sakha et al. (pt. 3) [36]	2004	M, 11 m	Iran	RSV	LRTI	Myo.	Reduced LV function, reduced contractility	AW, IF	NA	Residual Disease	6
Salfi et al. [95]	2024	F, 6 m	Italy	RSV	LRTI	Myo.	Negative	AW, RT-PCR	Neg.	Death	6
Schnaubelt et al. [96]	2022	M, 74 y	Austria	RSV	LRTI	Myo.	Highly reduced LV function and bilateral pleural effusion	AW, RT-PCR	NA	Death	3
Sunkonkit et al. [120]	2019	F, 10 y	Thailand	hMPV	LRTI	RD	NA	AW, RT-PCR	NA	Recovery	3
Taira et al. (Kawashima et al. 2021, pt. 21) [79]	1983	M, 58 y	Japan	RSV	Neg.	RD	NA	Serum	NA	Recovery	4
Taira et al. (Kawashima et al. 2021, pt. 22) [79]	1983	M, 59 y	Japan	RSV	LRTI	RD	Reduced LV function	Serum	NA	Residual Disease	6
Takamizawa et al. [121]	2022	F, 10 m	Japan	hMPV	LRTI	Myo.	Depressed LV function Takotsubo	AW, RT-PCR	Neg.	Recovery	6
Thomas et al. (pt. 2) [97]	1997	M, 3 m	USA	RSV	LRTI	Myo.	Dilated LV	AW, RT-PCR	NA	Recovery	6
Thomas et al. (pt. 1) [97]	1997	F, 3 m	USA	RSV	LRTI	Myo.	Reduced LV function	AW, RT-PCR	NA	Recovery	6
Wang et al. [122]	2024	M, 68 y	Taiwan	hMPV	LRTI	Myo.	LV function highly depressed (15%), heart failure with stress cardiomyopathy and myocarditis	AW, RT-PCR	NA	Death	6
Weinreich et al. [123]	2015	M, 25 y	USA	hMPV	URTI	Myo.	Severely depressed LV function, severely enlarged left atrial volume and dilated LV	AW, RT-PCR	NA	Recovery	6
Xu et al. [98]	2018	F, 24 m	China	RSV	LRTI	Myo.	Negative	AW, RT-PCR	NA	Death	6
Yakut et al. [124]	2020	M, 2.5 m	Turkey	hMPV	LRTI	Myo.	Depressed ejection fraction, severely dilated LV	AW, RT-PCR	NA	Recovery	6
Yamamoto et al. [99]	2013	M, 2 y	Japan	RSV	LRTI	RD	Normal	AW, RT-PCR	NA	Death	6

**Table A3:** Qualitative assessment of the case reports included in the present systematic review in accordance with Murad et al. [54]. D1: “Does the patient(s) represent(s) the whole experience of the investigator (center) or is the selection method unclear to the extent that other patients with similar presentation may not have been reported? D2: “Was the exposure adequately ascertained?”; D3: “Was the outcome adequately ascertained?”; D4: “Were other alternative causes that may explain the observation ruled out?”; D7: “Was follow-up long enough for outcomes to occur?”; D8: “Is the case(s) described with sufficient details to allow other investigators to replicate the research or to allow practitioners to make inferences related to their own practice?”. Each item was scored independently: “yes” provided a +1 to the sum score, while “no” granted no additional score (potential range 0 to 6).

Study	D1	D2	D3	D4	D7	D8	TOT
Abdulghani et al. [58]	yes	yes	yes	yes	yes	yes	6
Al Hennawi et al. (pt. 1) [100]	yes	yes	yes	yes	yes	yes	6
Al Hennawi et al. (pt. 2) [100]	yes	yes	yes	yes	yes	yes	6
Almajed et al. [59]	yes	yes	yes	yes	yes	yes	6
Anstett et al. [101]	yes	yes	yes	yes	yes	yes	6
Araki et al. (Kawashima et al., pt. 2) [79]	yes	no	yes	no	no	yes	3
Armstrong and Menahem [60]	yes	yes	yes	yes	yes	yes	6
Aydogan et al. [61]	yes	yes	yes	yes	yes	yes	6
Bairan et al. [62]	yes	no	yes	no	yes	yes	4
Bhatia et al. [102]	yes	yes	yes	yes	yes	yes	6
Campbell et al. [63]	yes	yes	yes	yes	yes	yes	6
Castillo-Morales et al. [103]	yes	yes	yes	yes	yes	yes	6
Choi et al. [104]	yes	yes	yes	yes	yes	yes	6
Dabbah et al. [64]	yes	yes	yes	yes	yes	yes	6
David et al. [105]	yes	yes	yes	yes	yes	yes	6
Donnerstein et al. (pt. 1) [65]	yes	yes	yes	yes	yes	yes	6
Donnerstein et al. (pt. 2) [65]	yes	yes	yes	yes	yes	yes	6
Donnerstein et al. (pt. 4) [65]	yes	yes	yes	yes	yes	yes	6
Donnerstein et al. (pt. 5) [65]	yes	yes	yes	yes	yes	yes	6
Donnerstein et al. (pt. 7) [65]	yes	yes	yes	yes	yes	yes	6
Donnerstein et al. (pt. 8) [65]	yes	yes	yes	yes	yes	yes	6
Eng et al. [66]	yes	yes	yes	yes	yes	yes	6
Erdogan et al. [37]	yes	yes	yes	yes	yes	yes	6
Fox et al. [67]	yes	yes	yes	yes	yes	yes	6
Garcia Suarez et al. [68]	yes	yes	yes	yes	yes	yes	6
Garrido Ocaña et al. (pt. 1) [69]	yes	yes	yes	yes	yes	yes	6
Garrido Ocaña et al. (pt. 2) [69]	yes	yes	yes	yes	yes	yes	6
Gavotto et al. [70]	yes	yes	yes	yes	yes	yes	6
Giles and Gohd (pt. 1) [71]	yes	yes	yes	yes	yes	yes	6
Giles and Gohd (pt. 2) [71]	yes	yes	yes	yes	yes	yes	6
Gopalan et al. [107]	yes	no	yes	no	no	yes	3
Goto et al. (Kawashima et al., pt. 12) [79]	yes	yes	yes	yes	yes	yes	6
Haddad et al. [72]	yes	yes	yes	yes	yes	yes	6
Huang et al. [73]	yes	yes	yes	yes	yes	yes	6
Hutchison et al. [74]	yes	yes	yes	yes	yes	yes	6
Imamura et al. [75]	yes	yes	yes	yes	yes	yes	6
Ingram et al. [108]	yes	no	yes	no	no	yes	3
Ishikawa et al. (Kawashima pt. 10) [79]	yes	yes	yes	yes	yes	yes	6
Jaber et al. [76]	yes	yes	yes	yes	yes	yes	6
Jasti et al. [77]	yes	yes	yes	yes	yes	yes	6
Jingu et al. [109]	yes	yes	yes	yes	yes	yes	6
Jiun et al. [110]	yes	yes	yes	yes	yes	yes	6
Kaburaki et al. [111]	yes	yes	yes	yes	yes	yes	6
Kakimoto et al. (pt. 1) [78]	yes	yes	yes	yes	yes	yes	6
Kakimoto et al. (pt. 2) [78]	yes	yes	yes	yes	yes	yes	6
Karatza et al. [28]	yes	no	yes	no	no	yes	3
Kawashima et al. (pt. 11) [79]	yes	no	yes	no	no	yes	3
Kawashima et al. (pt. 14) [79]	yes	no	yes	no	no	yes	3
Kawashima et al. (pt. 5) [79]	yes	no	yes	no	no	yes	3
Kawashima et al. (pt. 7) [79]	yes	no	yes	no	no	yes	3
Kawashima et al. (pt. 8) [79]	yes	yes	yes	yes	yes	yes	6
Kayiran et al. [80]	yes	yes	yes	yes	yes	yes	6
Kongala et al. [112]	yes	yes	yes	yes	yes	yes	6
Krivonos et al. [113]	yes	yes	yes	yes	yes	yes	6
Lademann et al. [114]	yes	yes	yes	yes	yes	yes	6
Makhlouf et al. [115]	yes	yes	yes	yes	yes	yes	6
Mehta et al. [116]	yes	no	no	no	yes	yes	3
Menahem and Uren [82]	yes	yes	yes	yes	yes	yes	6
Menahem S [81]	yes	yes	yes	yes	yes	yes	6
Menchise A [83]	yes	yes	yes	yes	yes	yes	6
Mihyawi et al. [117]	yes	yes	yes	yes	yes	yes	6
Milas et al. [38]	yes	yes	yes	yes	yes	yes	6
Misirlioglu et al. [84]	yes	yes	yes	yes	yes	yes	6
Miura et al. [40]	yes	no	yes	no	no	yes	3
Mizuno et al. (Kawashima et al., pt. 13) [79]	yes	yes	yes	yes	yes	yes	6
Monteiro de Almeida Magalhaes et al. [85]	yes	yes	yes	yes	yes	yes	6
Mustafa et al. [86]	yes	yes	yes	yes	yes	yes	6
O’Brien et al. [39]	yes	yes	yes	yes	yes	yes	6
Olesch and Bullock [87]	yes	no	yes	no	yes	yes	4
Oulego-Erroz et al. [88]	no	yes	yes	yes	no	no	3
Owensby et al. [118]	yes	yes	yes	yes	yes	yes	6
Patel et al. [89]	yes	yes	yes	yes	yes	yes	6
Playfor and Khader [90]	yes	no	no	no	yes	yes	3
Puchkov et al. [91]	yes	no	no	yes	yes	yes	4
Rubach et al. [41]	yes	no	no	yes	yes	yes	4
Ramzali et al. (pt. 1) [92]	yes	no	no	yes	yes	yes	4
Ramzali et al. (pt. 3) [92]	yes	no	no	yes	yes	yes	4
Ramzali et al. (pt. 4) [92]	yes	no	no	yes	yes	yes	4
Ramzali et al. (pt. 6) [92]	yes	yes	yes	yes	yes	yes	6
Ramzali et al. (pt. 7) [92]	yes	yes	yes	yes	yes	yes	6
Raphael et al. [93]	yes	yes	yes	yes	yes	yes	6
Raphael et al. [93]	yes	yes	yes	yes	yes	yes	6
Rethnaswamy et al. [125]	yes	yes	yes	no	yes	yes	5
Rodriguez-Nava et al. [119]	yes	yes	yes	yes	yes	yes	6
Royzman et al. [94]	yes	no	yes	no	no	yes	3
Rubach et al. [41,95]	yes	no	yes	no	no	yes	3
Sakha et al. (pt. 1) [36]	yes	no	yes	no	no	yes	3
Sakha et al. (pt. 2) [36]	yes	yes	yes	yes	yes	yes	6
Sakha et al. (pt. 3) [36]	yes	yes	yes	yes	yes	yes	6
Salfi et al. [95]	yes	yes	yes	yes	yes	yes	6
Schnaubelt et al. [96]	yes	no	yes	no	no	yes	3
Sunkonkit et al. [120]	yes	no	yes	no	no	yes	3
Taira et al. (Kawashima et al. 2021, pt. 21) [79]	yes	no	yes	no	yes	yes	4
Taira et al. (Kawashima et al. 2021, pt. 22) [79]	yes	yes	yes	yes	yes	yes	6
Takamizawa et al. [121]	yes	yes	yes	yes	yes	yes	6
Thomas et al. (pt. 2) [97]	yes	yes	yes	yes	yes	yes	6
Thomas et al. (pt. 1) [97]	yes	yes	yes	yes	yes	yes	6
Wang et al. [122]	yes	yes	yes	yes	yes	yes	6
Weinreich et al. [123]	yes	yes	yes	yes	yes	yes	6
Xu et al. [98]	yes	yes	yes	yes	yes	yes	6
Yakut et al. [124]	yes	yes	yes	yes	yes	yes	6
Yamamoto et al. [99]	yes	yes	yes	yes	yes	yes	6

**Note:** Items D5 and D6 from the original model of Murad et al. [54] were removed as specifically designed for interventional case reports including data on the pharmacological treatment and drug side effects/interactions.

**Table A4:** Comorbidities associated with included cases of human metapneumovirus (hMPV) and respiratory syncytial virus (RSV) infections. Comparisons were performed by means of chi-squared test.

Comorbidity	hMPV (n/31, %)	RSV (n/71, %)	*p*-Value
Prematurity	0, -	4, 5.6%	0.427
Underlying genetic syndrome	0, -	2, 2.8%	0.867
Heart disease (any)	0, -	9, 12.7%	0.090
History of myocardial infarction	2, 6.5%	1, 1.4%	0.454
History of congestive heart disease	1, 3.2%	3, 4.2%	1.000
History of peripheral vascular disease	2, 6.5%	1, 1.4%	0.454
History of cerebrovascular disease	1, 3.2%	0, -	0.668
History of chronic lung disease	4, 12.9%	6, 8.5%	0.739
History of rheumatic disease	0, -	1, 1.4%	1.000
History of diabetes	4, 12.9%	4, 5.6%	0.209
History of chronic kidney disease	4, 12.9%	1, 1.4%	0.048
History of chronic liver disease	1, 3.2%	1, 1.4%	0.609
Previous solid malignancies	2, 6.5%	1, 1.4%	0.454
Previous blood malignancies	1, 3.2%	3, 4.2%	0.811
Blood Marrow transplant	0, -	1, 1.4%	1.000

**Table A5:** Results of multivariable analysis. Four distinct models were designed using binary logistic regression and included the diagnosis of hMPV vs. RSV as the outcome variable. Explanatory variables were grouped into four a priori specified models, as follows: Model I included the main baseline characteristics of the patients: sex, age < 18 years at hospital admission, and Charlson Comorbidity Index (CCI); Model II focused on clinical presentation at hospital admission and included the variables entered in Model I together with fever, shortness of breath, dyspnea, chest pain, cough, sneezing, rhinorrhea, asthenia, convulsions, lethargy/altered mental status, any sign of arrhythmia, tachycardia, tachypnoea, hypotension, depressed ejection fraction (EF) on ultrasonography, and cardiac arrest; Model III focused on respiratory support and included the baseline variables from Model I together with high-flow oxygen therapy, continuous positive airway pressure (CPAP), mechanical ventilation, and extracorporeal membrane oxygenation (ECMO); Model IV focused on the main clinical outcomes and included the baseline variables from Model I together with diagnoses of myocarditis, pericarditis, rhythm abnormalities, heart failure, and myocardial infarction, as well as intensive care unit (ICU) admission and death. For ease of interpretation, *p*-values < 0.05 are highlighted in bold.

Variable	Model I (Background Features)	Model II (Clinical Features at Hospital Admission)	Model III (Need for Respiratory Support)	Model IV (Main Outcome Variables)
	aOR (95% CI)	aOR (95% CI)	aOR (95% CI)	aOR (95% CI)
Demographic features				
Male gender	0.670 (0.261; 1.719)	0.814 (0.206; 3.219)	0.875 (0.311; 2.466)	0.778 (0.253; 2.396)
Age < 18 years at hospital admission	0.328 (0.103; 1.049)	0.378 (0.046; 3.080)	0.317 (0.091; 1.109)	0.376 (0.093; 1.524)
Charlson Comorbidity Index (CCI)				
Mild (CCI 1 to 2)	2.855 (0.866; 9.414)	3.141 (0.547; 18.044)	3.072 (0.877; 10.761)	3.877 (0.979; 15.356)
Moderate (CCI 3 to 4) to Severe (CCI ≥ 5)	2.312 (0.541; 9.882)	3.337 (0.384; 28.979)	2.554 (0.543; 12.021)	3.713 (0.654; 21.095)
Clinical features				
Fever (temperature ≥ 38.0 °C)		4.325 (0.704; 26.569)		
Shortness of breath		7.901 (0.776; 80.466)		
Dyspnea		**16.280 (1.370; 193.475)**		
Chest pain		0.804 (0.168; 3.851)		
Cough		**6.059 (1.062; 34.552)**		
Sneezing		1.208 (0.157; 9.318)		
Rhinorrhea		**0.031 (0.003; 0.286)**		
Asthenia		0.291 (0.058; 1.466)		
Convulsions		20.461 (0.888; 471.489)		
Lethargy/Altered mental status		**0.103 (0.015; 0.712)**		
Any Arrhythmia		0.167 (0.019; 1.483)		
Tachycardia		2.015 (0.409; 9.920)		
Tachypnoea		1.193 (0.288; 4.935)		
Hypotension		0.926 (0.234; 3.659)		
Depressed EF at ultrasonography		2.659 (0.588; 12.036)		
Cardiac arrest		0.558 (0.080; 3.895)		
Need for respiratory support				
O_2_ high flow			1.437 (0.357; 5.783)	
CPAP			0.695 (0.186; 2.606)	
Mechanical Ventilation			1.975 (0.509; 7.665)	
ECMO			2.177 (0.486; 9.759)	
Outcome				
Myocarditis				2.389 (0.504; 11.311)
Pericarditis				1.628 (0.453; 5.848)
Any Rhythm anomaly				1.247 (0.379; 4.103)
Heart failure				0.800 (0.247; 2.587)
Infarction				0.695 (0.025; 19.686)
ICU admission				**11.033 (1.125; 108.173)**
Death				2.698 (0.722; 10.087)
