# Supplementary material for: Cardiac Complications of Infections Due to Respiratory Syncytial Virus and Human Metapneumovirus: A Systematic Review and Pooled Analysis of Clinical Features
Source: Viruses. 2026 Oct 7;18(10):1106. doi: 10.3390/v18101106 (PMC13709401; doi:10.3390/v18101106)
Supplement: Supplementary file 1 [file viruses-18-01106-s001.zip › Supplementary File S1 - PRISMA_2020_Checklist_RSV_hMPV.pdf]

PRISMA 2020 Checklist

Cardiac complications of infections due to Respiratory Syncytial Virus and human Metapneumovirus: a systematic review and pooled analysis of clinical features

Completed against the submitted manuscript version supplied by the authors

Section and topic	Item #	PRISMA 2020 checklist item	Location where item is reported
TITLE<br>Title	1	Identify the report as a systematic review.	Title, p. 1
ABSTRACT<br>Abstract	2	See the PRISMA 2020 for Abstracts checklist.	Abstract, p. 2
INTRODUCTION<br>Rationale	3	Describe the rationale for the review in the context of existing knowledge.	Introduction, pp. 2–3
INTRODUCTION<br>Objectives	4	Provide an explicit statement of the objective(s) or question(s) the review addresses.	Introduction, p. 3; Methods §2.1, pp. 3–4
METHODS<br>Eligibility criteria	5	Specify the inclusion and exclusion criteria for the review and how studies were grouped for the syntheses.	Methods §§2.1.2, 2.3–2.4, pp. 4–5; Appendix Table A2
METHODS<br>Information sources	6	Specify all databases, registers, websites, organisations, reference lists and other sources searched or consulted, and the date when each source was last searched or consulted.	Methods §2.2, p. 4 (PubMed, Scopus, EMBASE; snowball search; last search 14 Aug 2026)
METHODS<br>Search strategy	7	Present the full search strategies for all databases, registers and websites, including any filters and limits used.	Methods §2.2, Table 1, pp. 4–5
METHODS<br>Selection process	8	Specify the methods used to decide whether a study met the inclusion criteria, including how many reviewers screened each record/report and whether they worked independently.	Methods §2.3, p. 5. Full texts screened by two reviewers.
METHODS<br>Data collection process	9	Specify the methods used to collect data from reports, including how many reviewers collected data from each report, whether they worked independently, and any processes for obtaining/confirming data.	Methods §2.5, pp. 5–6. Extracted variables are described.
METHODS<br>Data items	10a	List and define all outcomes for which data were sought; specify whether all compatible results were sought and, if not, the methods used to decide which results to collect.	Methods §§2.1.2, 2.4–2.5, pp. 4–6; Appendix Tables A1–A2
METHODS<br>Data items	10b	List and define all other variables for which data were sought and describe assumptions made about missing or unclear information.	Methods §2.5, pp. 5–6; Appendix Tables A1–A2..
METHODS<br>Study risk of bias assessment	11	Specify the methods used to assess risk of bias in included studies, including tool(s), number of reviewers, independence, and resolution of disagreements.	Methods §2.6, p. 6 (Murad et al.; two independent reviewers; consensus/third reviewer)
METHODS<br>Effect measures	12	Specify for each outcome the effect measure(s) used in the synthesis or presentation of results.	Methods §2.7, pp. 6–7 (counts/percentages, means with 95% CI, aORs; Wilson intervals for cardiac manifestations reported in Results/Figure 5)
METHODS<br>Synthesis methods	13a	Describe the processes used to decide which studies were eligible for each synthesis.	Methods §§2.3–2.5, pp. 5–6; cases required individual-level characterization and laboratory-confirmed infection
METHODS<br>Synthesis methods	13b	Describe any methods required to prepare the data for presentation or synthesis, such as handling missing summary statistics or data conversions.	Methods §§2.4–2.5, pp. 5–6 (duplicate/cross-posted cases reconciled).
METHODS<br>Synthesis methods	13c	Describe any methods used to tabulate or visually display results of individual studies and syntheses.	Methods §§2.7–2.8, pp. 6–7; Tables 2–7, Figures 2–6 and Appendix figures/tables
METHODS<br>Synthesis methods	13d	Describe any methods used to synthesize results and provide a rationale for the choice(s). If meta-analysis was performed, describe the model, heterogeneity assessment and software.	Methods §2.7, pp. 6–7. Pooled individual-case descriptive/univariate analyses, logistic regression and MCA are described; no conventional meta-analysis of study-level effect estimates was performed.
METHODS<br>Synthesis methods	13e	Describe any methods used to explore possible causes of heterogeneity among study results.	Methods §2.7, p. 7 (exploratory MCA; age-stratified sensitivity analysis)
METHODS<br>Synthesis methods	13f	Describe any sensitivity analyses conducted to assess robustness of synthesized results.	Methods §2.7, p. 7 (age strata: <1 year, 1–17 years, ≥18 years)
METHODS<br>Reporting bias assessment	14	Describe any methods used to assess risk of bias due to missing results in a synthesis.	Not formally assessed. Reporting/publication bias is discussed as a limitation in §4.4, pp. 27–29.
METHODS<br>Certainty assessment	15	Describe any methods used to assess certainty (or confidence) in the body of evidence for an outcome.	Not reported / no formal certainty-of-evidence framework (e.g., GRADE) applied.
RESULTS<br>Study selection	16a	Describe the results of the search and selection process, from records identified to studies included, ideally using a flow diagram.	Results §3.1, pp. 7–8; Figure 1 (PRISMA flow diagrams)
RESULTS<br>Study selection	16b	Cite studies that might appear to meet the inclusion criteria but were excluded, and explain why they were excluded.	Reasons for exclusion are shown in Figure 1, p. 8; individual excluded reports are not cited.
RESULTS<br>Study characteristics	17	Cite each included study and present its characteristics.	Results §3.2, pp. 8–9; Appendix Table A2; included reports cited in §3.1
RESULTS<br>Risk of bias in studies	18	Present assessments of risk of bias for each included study.	Results §3.2, p. 9; Figure 3; Appendix Table A3
RESULTS<br>Results of individual studies	19	For all outcomes, present for each study summary statistics for each group and an effect estimate with precision where appropriate.	Individual case characteristics are provided in Appendix Table A2. Study-level effect estimates are generally not

			applicable because the evidence base consists predominantly of case reports/case series.
RESULTS<br>Results of syntheses	20a	For each synthesis, briefly summarise the characteristics and risk of bias among contributing studies.	Results §§3.2–3.3, pp. 8–18; Figure 3; Appendix Tables A2–A3
RESULTS<br>Results of syntheses	20b	Present results of all statistical syntheses conducted, including summary estimates and precision where applicable.	Results §§3.3–3.5, pp. 10–23; Tables 2–7; Figures 4–6. Wilson 95% intervals for major cardiac manifestations in §3.3.3/Figure 5.
RESULTS<br>Results of syntheses	20c	Present results of all investigations of possible causes of heterogeneity among study results.	Results §3.5, p. 22 (MCA); §3.6, p. 23 and Appendix Table A5 (age-stratified analyses)
RESULTS<br>Results of syntheses	20d	Present results of all sensitivity analyses conducted to assess robustness of synthesized results.	Results §3.6, p. 23; Appendix Table A5
RESULTS<br>Reporting biases	21	Present assessments of risk of bias due to missing results for each synthesis assessed.	No formal assessment reported. Selective/reporting bias is acknowledged in Discussion §4.4, pp. 27–29.
RESULTS<br>Certainty of evidence	22	Present assessments of certainty (or confidence) in the body of evidence for each outcome assessed.	Not reported / no formal certainty-of-evidence assessment performed.
DISCUSSION<br>Discussion	23a	Provide a general interpretation of the results in the context of other evidence.	Discussion §§4.1–4.3, pp. 23–27
DISCUSSION<br>Discussion	23b	Discuss any limitations of the evidence included in the review.	Discussion §4.4, pp. 27–29
DISCUSSION<br>Discussion	23c	Discuss any limitations of the review processes used.	Discussion §4.4, pp. 27–29 (case-report selection, reporting bias, retrospective/incomplete data, diagnostic heterogeneity, small sample and statistical imprecision)
DISCUSSION<br>Discussion	23d	Discuss implications of the results for practice, policy, and future research.	Discussion §4.3, pp. 25–27; §4.5, p. 29; Conclusions, p. 29
OTHER INFORMATION<br>Registration and protocol	24a	Provide registration information for the review, including register name and registration number, or state that the review was not registered.	Methods §2.1.1, pp. 3–4: PROSPERO CRD420251170706
OTHER INFORMATION<br>Registration and protocol	24b	Indicate where the review protocol can be accessed, or state that a protocol was not prepared.	Methods §2.1.1, p. 4; Supplementary File S1 / PROSPERO record
OTHER INFORMATION<br>Registration and protocol	24c	Describe and explain any amendments to information provided at registration or in the protocol.	Not reported.
OTHER INFORMATION<br>Support	25	Describe sources of financial or non-financial support for the review and the role of funders or sponsors.	Funding statement, p. 29: no external funding
OTHER INFORMATION<br>Competing interests	26	Declare any competing interests of review authors.	Conflicts of Interest statement, p. 29: authors declare no conflicts of interest
OTHER INFORMATION<br>Availability of data, code and other materials	27	Report which review materials are publicly available and where they can be found (e.g., extracted data, analysis data, code, forms, other materials).	Supplementary Materials and Data Availability Statement, p. 29: raw data available as supplementary material.

Source: PRISMA 2020 checklist) [43,44].
